# 3D printed protein-based robotic structures actuated by molecular motor assemblies

**DOI:** 10.1038/s41563-022-01258-6

**Published:** 2022-05-26

**Authors:** Haiyang Jia, Johannes Flommersfeld, Michael Heymann, Sven K. Vogel, Henri G. Franquelim, David B. Brückner, Hiromune Eto, Chase P. Broedersz, Petra Schwille

**Affiliations:** 1 Max Planck Institute of Biochemistry, Martinsried, Germany; 2 Arnold Sommerfeld Center for Theoretical Physics, Center for NanoScience, Ludwig-Maximilians-Universität München, Munich, Germany; 3 Department of Physics and Astronomy, Vrije Universiteit Amsterdam, Amsterdam, The Netherlands; 4 Institute of Biomaterials and Biomolecular Systems, University of Stuttgart, Stuttgart, Germany

**Keywords:** Synthetic biology, Supramolecular assembly, Biomaterials - proteins, Actuators, Biological physics

## Abstract

Upscaling motor protein activity to perform work in man-made devices has long been an ambitious goal in bionanotechnology. The use of hierarchical motor assemblies, as realized in sarcomeres, has so far been complicated by the challenges of arranging sufficiently high numbers of motor proteins with nanoscopic precision. Here, we describe an alternative approach based on actomyosin cortex-like force production, allowing low complexity motor arrangements in a contractile meshwork that can be coated onto soft objects and locally activated by ATP. The design is reminiscent of a motorized exoskeleton actuating protein-based robotic structures from the outside. It readily supports the connection and assembly of micro-three-dimensional printed modules into larger structures, thereby scaling up mechanical work. We provide an analytical model of force production in these systems and demonstrate the design flexibility by three-dimensional printed units performing complex mechanical tasks, such as microhands and microarms that can grasp and wave following light activation.

## Abstract (web summary)

Three-dimensional printed protein-based robotic structures are actuated by exoskeleton-like coats of molecular motor assemblies upon the spatially targeted release of chemical fuel, resulting in micrometre-scale shape-morphing activity.

## Main

Living systems are a source of inspiration for man-made robotics^1–3^ with regard to flexibility, scalability and resilience. Recent progress in micro-three-dimensional (micro-3D) printing and synthetic biology^4^ raises expectations that a bottom-up design of nano- to microscale biorobots^5,6^ directly using biomolecules may become reality. Cellular motor proteins^7–11^, which convert metabolic energy directly into mechanical work, represent promising candidates from nature to execute mechanical operations on soft materials^12^. In contrast to electromechanical and biohybrid actuators^13–17^, which require considerable effort to downscale to the nano- and microscale^3^, a key challenge to using molecular motors is to upscale force and work to operate devices that are many orders of magnitude larger^18–21^. Efficient large-scale use of such protein motor systems for the actuation of soft robots has so far been mainly realized through cyborg constructions, hybridizing whole living muscle cells and tissue with soft polymer materials such as silicone^13,14,22^. Recently, a large-scale microtubule and kinesin motor-based active network reminiscent of stress fibres was successfully engineered that could be directly assembled in solution from light-activatable parts^23^.These untethered actuating elements produced forces in the micronewton range. However, to produce defined robotic structures with tethered actuators that more closely resemble biological motorized systems, two challenges must be met: the design of biomimetic templates that are both sufficiently malleable and physiologically compatible with the large-scale operation of motor protein systems, and the site-specific attachment of protein motors and networks to support complex mechanical operations.

## Concept of exoskeleton-actuated robotic structures

**Fig. 1:**
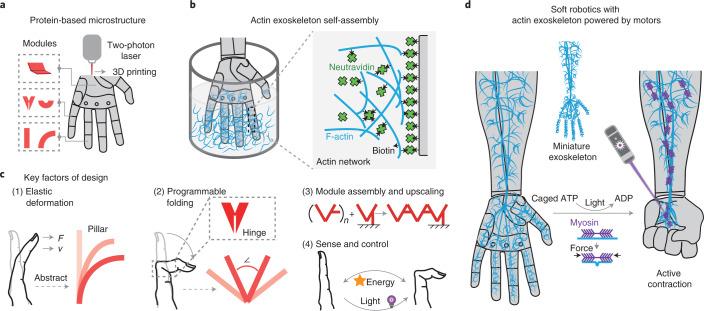
Key design concepts of protein-based soft robotic structures with an exoskeleton powered by molecular motors. **a**, Three-dimensional protein hydrogel printing procedure. **b**, Coupling of an actomyosin exoskeleton to the scaffold. **c**, Bioinspired design modules for programming and upscaling the protein-based soft robotics. *F* and *v* denote the active force and contraction velocity. **d**, Soft robotic arm powered by light-triggered molecular motor activity on the miniature exoskeleton.

To address the challenges outlined above, we introduce here shape-morphing robotic structures assembled from protein-based modular units^24,25^ (Fig. 1a) that are coated with, and actuated by, a minimal actomyosin exoskeleton. The protein-based units are generated by micro-3D printing with tunable mechanical properties and geometries that can be operated in very defined ways. The motorized exoskeleton can be viewed as a scaled-up actomyosin cortical layer^26^ connecting the different parts of the deformable robotic structure through specific non-covalent interactions (Fig. 1b). Our exoskeleton design harnesses the contractility generated by myosin molecular motors and transduces the resulting active stresses to perform large-scale mechanical work on the three-dimensional (3D) soft device without the need for hierarchically structured motor assemblies as in sarcomeres. We can characterize force production by the contractions of actomyosin layers and the resulting deformation of geometrically simple elementary units. We demonstrate that this approach can be applied to more complex shape-morphing structures by combining basic modules, such as pillars, panels and hinges, thereby mimicking key elements of, for example, human hands (Fig. 1c). Finally, we show that the exoskeleton design can be extended and scaled up to realize complex programmable shape transformations of active mechanical devices, and further achieve multistage functions of soft robotic elements with (selectively) light-activatable modules (Fig. 1d).

## Characterizing exoskeleton contractility

**Fig. 2:**
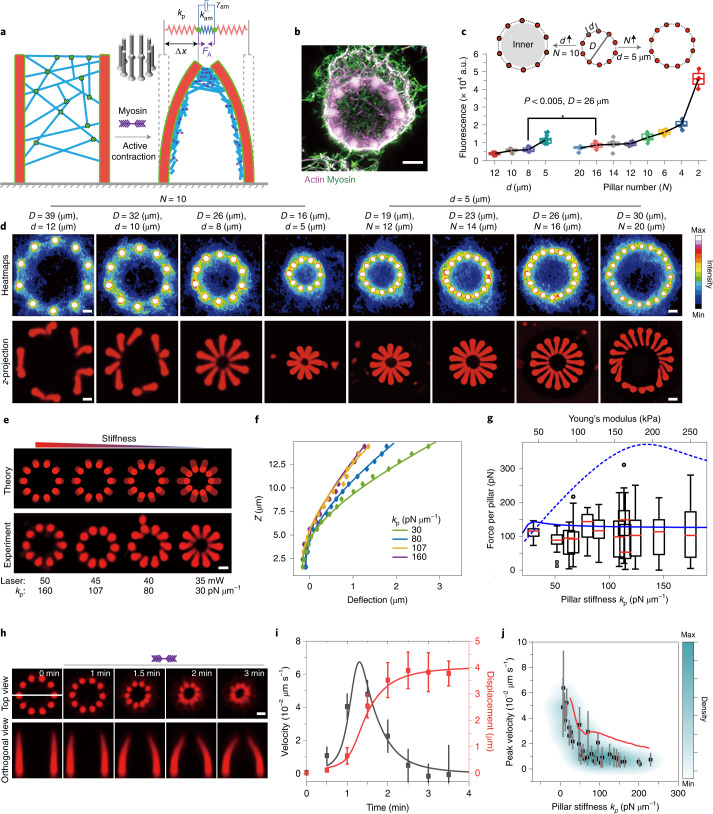
Pillar-based model system to quantify force production by contractile cortices on BSA hydrogel structures. **a**, Scheme depicting the coupling of an actomyosin exoskeleton to 3D printed protein hydrogel pillars. *F*_*A*_ denotes the active force exerted onto the pillars and *x* is the position of the pillar tips. *k*_*p*_ and *𝑘*_*am*_ denote the pillar stiffness and the spring constant of the actomyosin network, respectively. *γ*_*am*_ is the friction coefficient of the actomyosin network. **b**, The *z*-projection of the actomyosin exoskeleton on a pillar ring. Scale bar, 5 µm. **c**, Fluorescence intensity of an Alexa Fluor 647 phalloidin-labelled actin network on pillar rings with different pillar diameters *D* and interpillar distances *d*. The intensities are averages of the inner ring areas, shown in grey in the scheme (*n* = 8). *P* = 0.0032, analysis of variance (ANOVA) one-way statistical test. **d**, Fluorescence heatmaps and *z*-projections of actin networks on pillar rings with different sizes and pillar distances. The *z*-projections illustrate the contracted state of the pillar rings. Scale bars, 5 µm. The heatmaps demonstrate the averaged fluorescence intensity maps of actin networks imaged by confocal microscopy (*n* = 8). The Min and Max levels of the colour bar are the same for all images. **e**, Theoretical and measured (*z*-projection) contraction of pillar rings with different stiffness. Scale bar, 5 µm. For the predicted pillar profiles, the force measured for the stiffest pillar was used (*F*_A_ = 115 pN). **f**, Experimentally measured deflection (diamonds) and theoretical fits (lines) for pillars with different stiffnesses (same stiffnesses as in **e**). *Z* denotes the height of the pillar. **g**, Active force per pillar exerted on pillar rings with different stiffnesses (*n* = 10). The blue lines represent theoretical predictions of the generated active force with a purely density-dependent contractility (dashed) and with the myosin binding contractility model (solid). **h**, Contractile dynamics of the pillar ring in response to motor activation. Scale bar, 5 µm. The white line in the top left image indicates the position for generating orthogonal view. **i**, Pillar tip displacement and velocity during contraction (*k*_p_ = 35 pN μm^–1^). The experimental data (points) are fitted by the myosin binding contractility model (solid lines). **j**, Maximal velocity versus pillar stiffness. The data in **i** and **j** are shown as mean ± s.d.; *n* = 10 pillars were analysed for each data point. The solid red line in **i** and **j** represents the theoretical prediction. Box plots in **c** and **g**: the lines represent medians, box limits represent quartiles 1 and 3, whiskers represent 1.5 × interquartile range and points are outliers. The red fluorescence in **b**, **d** and **e** arise from the hydrogel itself; the reconstituted actomyosin networks are not shown in the figure. Source data

**Extended Data Fig. 1:**
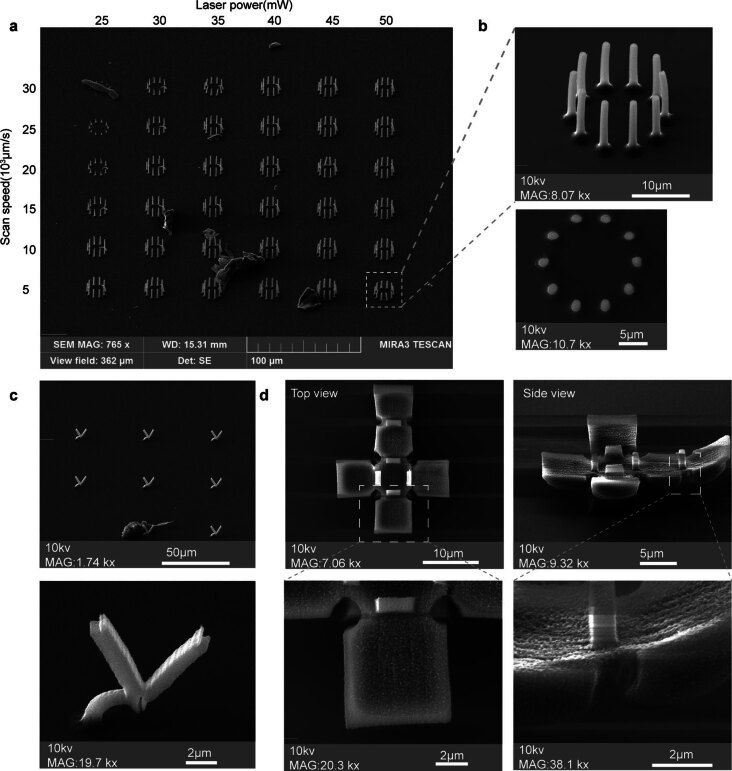
Scanning electron microscope images of 3D BSA microstructures. **a**.) Overview of the pillar ring array printed with varying laser powers and scan speeds. **b**.) Side view and top view of pillar ring. **c**.) Freestanding V units. **d**.) Self-folding hydrogel cube.

**Extended Data Fig. 2:**
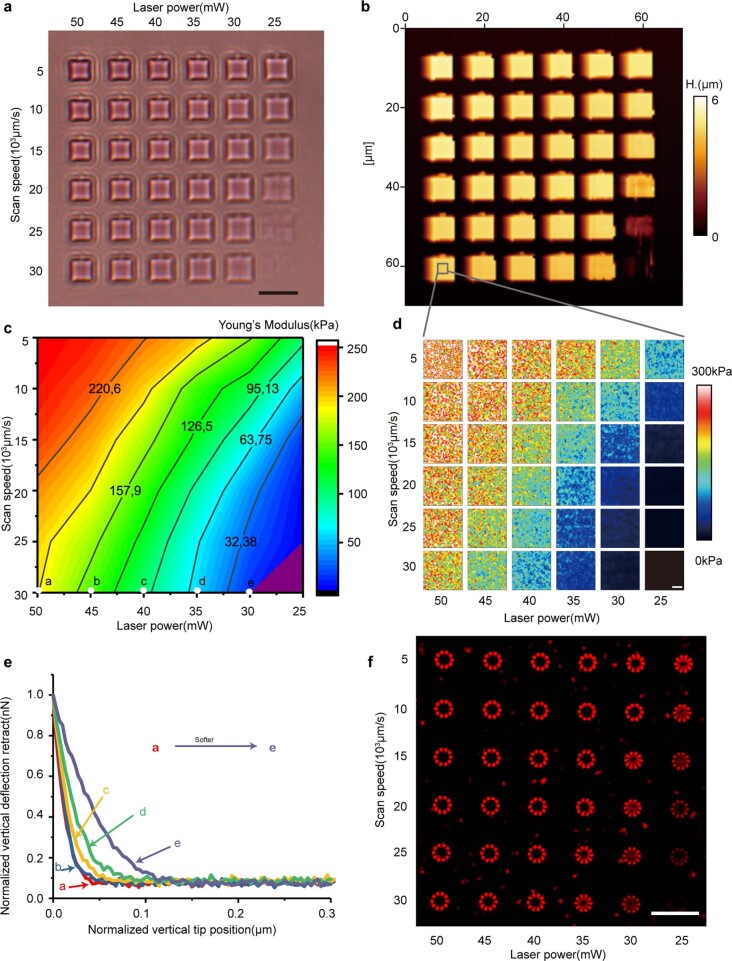
Young’s modulus measurement of the BSA microstructures by the Atomic Force Microscope (AFM). **a**.) The wide field imaging of BSA hydrogel structure (5x5x5 µm) array fabricated at different laser powers (25 mW-50 mW) and scan speeds (50000-300000 µm/s). Scale bar, 10 µm. **b**.) AFM imaging of the BSA hydrogel structure array. Color calibration bar represents the height of the microstructures. **c**.) Young’s modulus map of the hydrogel structures by varying printing parameters. **d**.) Heatmaps of AFM imaging. Color calibration bar represents the Young’s modulus of the microstructures. **e**.) Force curve of BSA structures with different elastic properties. **f**.) Z projection of the deformed pillar rings with different stiffness. Scale bar, 50 µm. Source data

**Extended Data Fig. 3:**
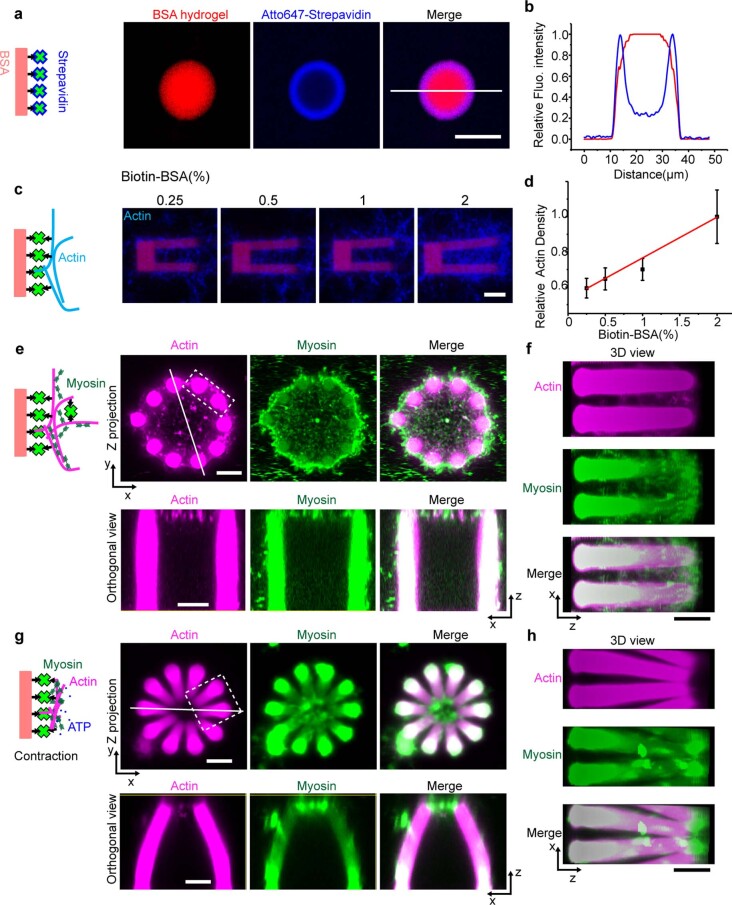
Coupling actin-myoin networks on 3D printed structures. **a**.) Fluorescence imaging and **b**.) line plot profile of pillar coated with Atto647-Streptavidin. Scale bar, 20 µm. The structure was fabricated with 1 mol% biotin-BSA, and then incubated with 1 mg/ml Atto647 labeled streptavidin. The white line in a. represents the position of the line plot profile. **c**.) Representative fluorescence images of micropillars coated with actin filaments. Structures were printed with varied biotin-BSA fraction from 0.25 mol% to 2 mol%. Scale bar, 5 µm. **d**.) Plot of relative actin density on pillars determined from the fluorescence intensity of Alexa Fluor 647 phalloidin labeled actin between the two pillars. Data points are shown as mean ± s.d. (n = 8). **e**,**f**.) Representative fluorescence images of actin-myosin networks on pillar rings. e.) Projections of z stacks and orthogonal views of the actin network. Actomyosin network mainly formed on the top and surface of the pillar ring. Scale bar, 5 µm. f.) 3D view of the actin network between adjacent pillars. Scale bar, 5 µm. **g**,**h**.) Representative fluorescence images of the contracted actin-myosin networks on pillar rings. g.) Z projection of and orthogonal views of the contracted actin-myosin network. Scale bar, 5 µm. h.) 3D view of the contracted actin network between adjacent pillars. Scale bar, 5 µm. Actin networks shown in e-h were crosslinked with 2.25 nM neutravidin. 10 % Alexa Fluor 488 labeled myosin was used for the imaging. Source data

**Extended Data Fig. 4:**
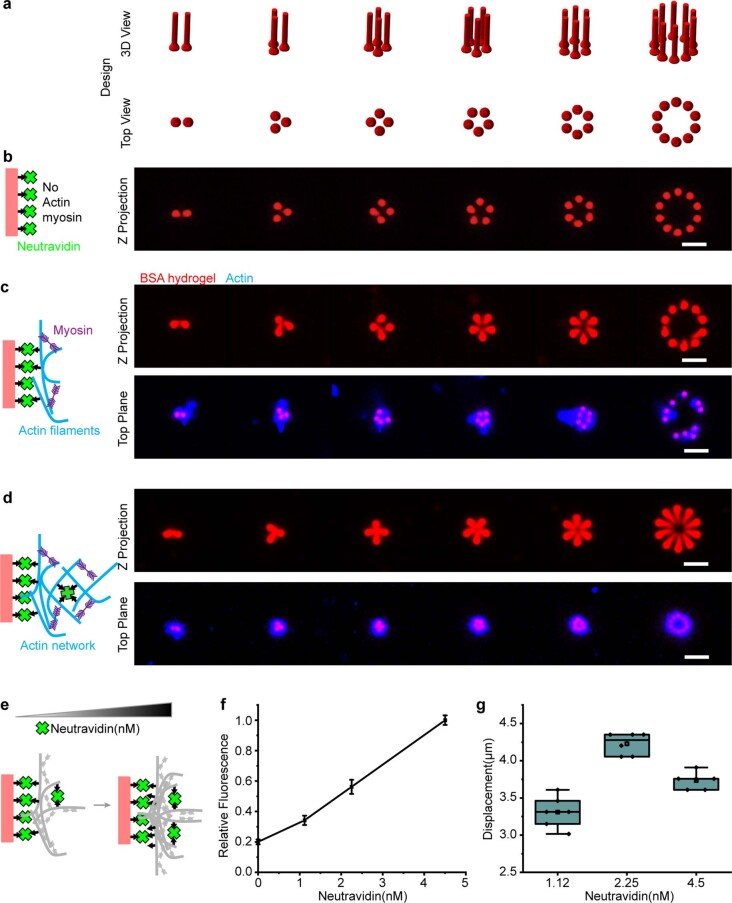
Cross-linked actin networks promote contraction integrity. **a**.) Schematic designs of pillar rings with different number of pillars. **b**.) Z projection of pillar rings without actin and myosin network. Scale bar, 10 µm. **c**.) Contraction of pillar rings coated with actin filaments. Scale bar, 10 µm. Pillar contraction started to differentiate on the ring with 10 pillars. **d**.) Contractions of pillar rings coated with actin network that was cross-linked by neutravidin (2.25 nM). Scale bar, 10 µm. Compared with the pillar rings coated with the actin filaments, the neutravidin cross-linked actin network could enhance the contraction integrity of pillar rings. **e**-**g**.) Neutravidin concentrations influence f.) the actin network density and g.) the contractions of pillar rings. Plot in f. demonstrates the relative fluorescence intensity of Alexa Fluor 647 phalloidin labeled actin networks (inner ring areas) that are crosslinked with different concentration of neutravidin. Data points are shown as mean ± s.d. (n = 6). The concentrations of the cross-linker neutravidin were titrated from 1.12 nM (1:40), 2.25 nM (1:20), to 4.5 nM (1:10) in order to optimize the contractility of actomyosin on the printed microstructures. The densities of actin networks on pillar rings were increased when increasing cross-linker concentrations. However, a reverse trend of pillar displacements was noticed in g. when the neutravidin concentration was increased to 4.5 nM, meaning the contraction was inhibited by the high cross-linking degree (neutravidin: biotin-actin ratio of 1:10 in our system). As such, we chose the 2.25 nM neutravidin concentration for further experiments, as this cross-linking condition best contractility was observed. Box plot in g: lines are median, box limits are quartiles 1 and 3, whiskers are 1.5×interquartile range and points are outliers. Source data

To quantitatively understand the basic performance of our contractile motorized exoskeleton with regard to force generation and transduction in the shape-morphing of 3D protein-based modules, we first designed a simple structure consisting of a ring of ten soft pillars (Fig. 2a and Extended Data Fig. 1a,b). The hydrogel pillar units were printed by two-photon polymerization of a bioresin consisting of bovine serum albumin (BSA) and rose bengal as photoinitiator. The Young’s modulus of the protein hydrogel can be tuned from 10 to 250 kPa (Extended Data Fig. 2), corresponding to the moduli of biological tissues^27,28^. Subsequently, the ring structure was biotinylated and decorated with an actin filament (F-actin) meshwork by biotin–actin and neutravidin coupling. The actin filaments were further crosslinked by neutravidin to enhance the network’s mechanical integrity (Fig. 2b and Extended Data Figs. 3 and 4). To complete the exoskeleton-like minimal actomyosin cortex, myofilaments pre-assembled with skeletal muscle myosin II were included. Upon ATP addition, these myosin motors generated contractile forces^26,29^ that were transduced by the exoskeleton, resulting in large-scale active stresses that drive the inward deflection of the pillar ring (Fig. 2 and Supplementary Video 1).

**Extended Data Fig. 5:**
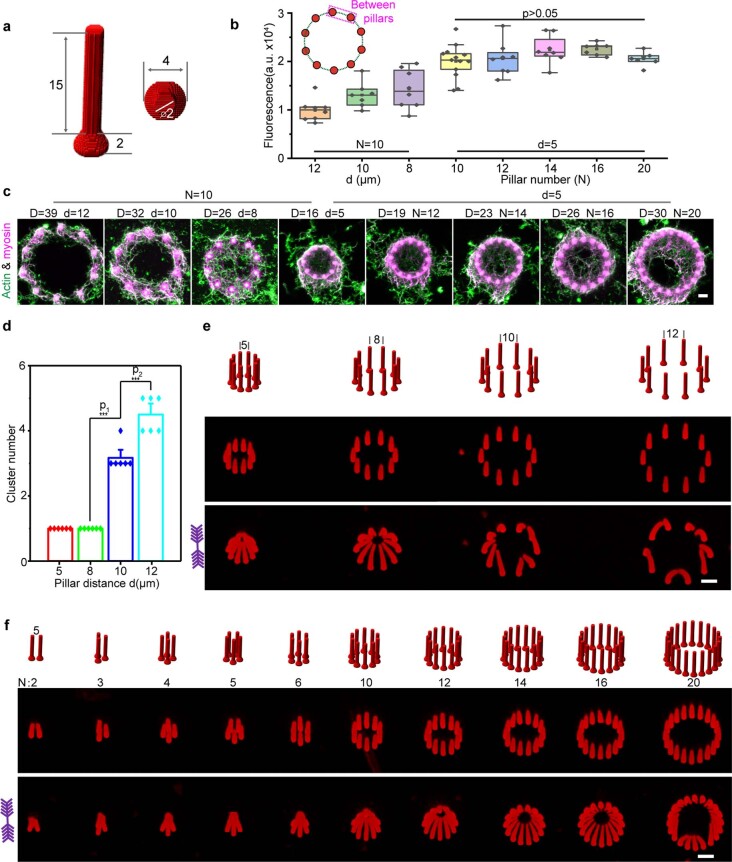
Contractile performance of the 3D structures depends on their dimensions. **a**.) Schematic design of pillar. The dimensions in this figure are given in micrometers. **b**.) Fluorescence intensity of Alexa Fluor 647 phalloidin labeled actin networks between two adjacent pillars on the pillar rings with different diameters (D, µm) and pillar distances (d, µm) (n = 8). The actin intensity increases with decreasing the adjacent pillar distances. When keeping the adjacent pillar distance constant and increasing the pillar number, the actin density between the adjacent pillars remains roughly constant. p = 0.223, Analysis of Variance (ANOVA) one-way statistical test. Box plot in b: lines are median, box limits are quartiles 1 and 3, whiskers are 1.5×interquartile range and points are outliers. **c**.) Representative images (z-projection) of the actin networks on pillar rings with different sizes and pillar distances. Scale bar, 5 µm. **d**.) The figure shows pillar clusters number versus pillar distance, which are corresponding to the results in e. The bar chart in d. is a variation of the box chart whose upper line represents Mean value and error bar represents the data range within 1.5x interquartile range (n = 6). ***p < 0.0005 (p_1_ = 1.37×10^−7^; p_2_ = 7.45×10^−4^), Analysis of Variance (ANOVA) one-way statistical test. **e**.) Representative 3D images of the deformed pillar rings with different pillar distances (N = 10). Scale bar, 5 µm. **f**.) Representative 3D images of the deformed pillar rings with different diameters (d = 5 µm). Scale bar, 5 µm. Pillar number was kept constant at 10 in e. Distance of nucleation sites was fixed at 5 µm in f. Printing parameters: laser power, 35 mW; scan speed, 30000 μm/s, resulting in Young’s modulus 57 kPa. Source data

Next, we investigated how the contractile performance of the 3D structures depends on their dimensions by varying the diameter *D* of the pillar ring. The diameter can be enlarged by increasing the interpillar distance *d* and maintaining a constant pillar number *N* (*N* = 10), or by increasing *N* and keeping *d* constant (*d* = 5 µm; Fig. 2c,d and Extended Data Fig. 5). We noticed that the deflection of pillars towards the centre was determined by the density and integrity of the actin mesh located inside the ring (Fig. 2c,d). Rings with larger diameters develop holes in the actomyosin meshwork, resulting in asymmetric and incoherent contraction of the ring as a whole (Fig. 2d). This effect is exacerbated by larger interpillar distance (Fig. 2d, left, and Extended Data Fig. 5c–e). In contrast, increasing the number of pillars in rings of similar diameter (*D* = 26 µm) results in significantly higher network density (Fig. 2c), better preserving the integrity of the networks. However, isotropic contractions were rarely observed for pillar rings with diameters ≥30 µm (Fig. 2d). These insights led us to a key design feature with regard to the scale over which the exoskeleton scaffold can achieve effective and coherent contractions, suggesting that concatenating smaller force-transducing modules could be a good option for engineering larger device designs.

**Extended Data Fig. 6:**
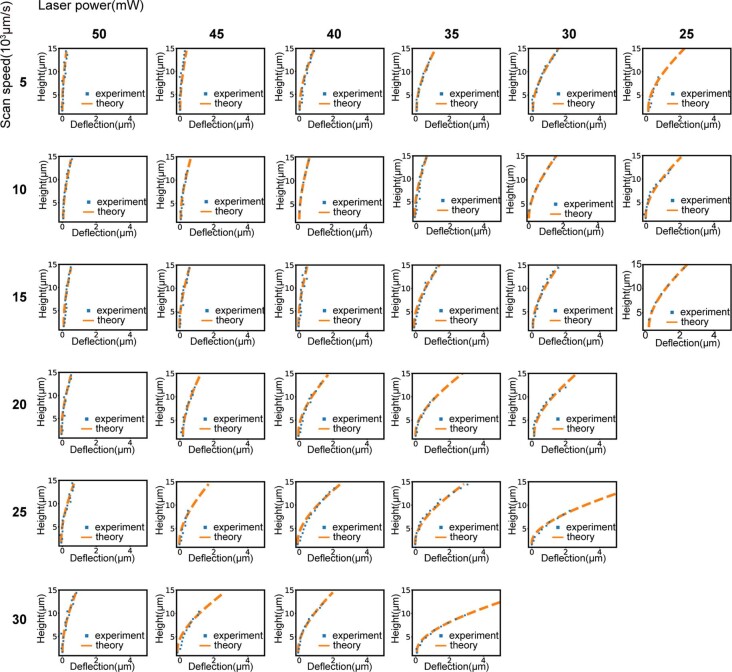
**Measured pillar deflection (blue point) and theoretical fitting (orange dash line) of the deformed pillars with different stiffness.** Source data

**Extended Data Fig. 7:**
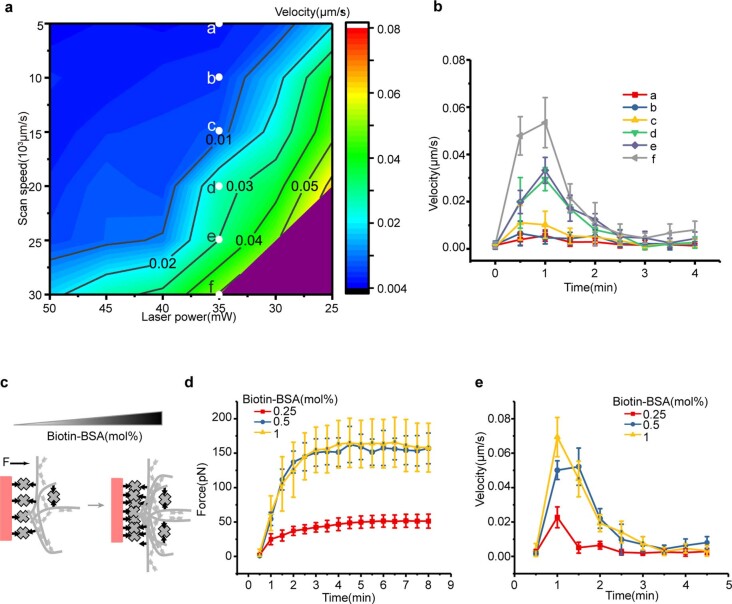
Contraction dynamics of pillar rings under active force. **a**,**b**) Stiffness of materials determines the contraction dynamics of pillar rings under active force. a.) The maximal motion velocity map of the pillar rings under active force. b.) Time course of motion velocity during contraction. c-e.) Actin network density influence the contraction dynamics of pillar rings. d.) Dynamics of active force generation on the pillar rings printed with different concentration of Biotin-BSA. e.) Time course of motion velocity during contraction. Data points in b-e are shown as mean ± s.d (n = 10). All structures used in d-e were printed with laser power 35 mW and scan speed 30000 μm/s, resulting in Young’s modulus 57 kPa. Source data

Another design feature of robotics engineering is to combine modules with different mechanical properties and programmable macroscopic deformations^30^. To explore the effect of elasticity on the contraction capacity of the actomyosin exoskeleton, we varied the stiffness of the pillars in the pillar ring test assay, while keeping the density of the meshwork constant (Fig. 2e,f and Extended Data Fig. 6). We found that the pillar deformations can be tuned by varying the material stiffness. The largest pillar deflection was observed in rings with a Young’s modulus of 57 ± 12 kPa (Extended Data Fig. 2f). In this case, the contractile cortex is able to reduce the radial dimension of the pillar ring at the tip by up to a factor of 0.5, resulting in large pillar shear strains of up to 0.3 (Fig. 2e). Importantly, the pillar ring design allows us to quantify the forces generated by the active contraction. By comparing the measured pillar deflection profiles using Euler beam theory^31^ (Fig. 2f and Supplementary Section 1), we measured the active forces generated by the actomyosin exoskeleton to be 126 ± 22 pN (*n* = 12) per pillar on full contraction. Individual myosin filaments under these conditions can generate forces close to 20–60 pN (refs. ^26,32,33^), suggesting that only a small fraction of the estimated (8.2 ± 2.0) × 10^3^ (mean ± s.d.; *n* = 9) myofilaments (Supplementary Fig. 1) in the exoskeleton effectively contribute to the contraction (Supplementary Section 2). However, the force generation in the system can be tuned by varying the density of the actin network coated on the 3D scaffolds by either changing the number of anchor points (Extended Data Figs. 3c,d and 7c,d) or by varying the crosslinker concentration (Extended Data Fig. 4e–g). For a given actin density, however, the force generation is robust over a wide range of pillar stiffnesses (Fig. 2g). The insensitivity of contractile forces to frame stiffness facilitates the predictability of force generation in more complex structures consisting of modules with different stiffnesses.

To understand the build-up of these generated forces, we next investigated the contraction dynamics of actomyosin networks on the microstructures. Upon myosin activation, the pillars start to bend inwards (Fig. 2h). The contraction velocity initially increases markedly over the course of approximately 1 min, followed by a decelerating contraction towards a final state (Fig. 2i). The maximal velocity that is reached during the contraction depends on pillar stiffness (Fig. 2j, Extended Data Fig. 7 and Supplementary Video 1). We observed a clear increase in peak velocity with decreasing pillar stiffness *k*_p_ below a value of 100 pN μm^–1^. Interestingly, the acceleration lasts much longer than the dynamics of single or spatiotemporally coordinated myosin motors in sarcomeres, which operate on the timescale of milliseconds (ref. ^34^).

## Mechanical feedback model for the contraction mechanism

To elucidate the mechanisms that underlie the observed robust force generation and dynamics of the contraction, we can describe our contractile system using a simple, one-dimensional (1D) analytical model. In this viscoelastic model, the elastic response of the pillar and network is represented by elastic springs and the viscous response of the network is characterized by a dashpot. The myosin activity is modelled by a time-dependent contractile force acting on the pillars (Fig. 2a, Supplementary Information and Supplementary Figs. 3–7). To capture the contraction dynamics, we included a microscopic description of force generation by the molecular motors: myosin filaments transiently bind to the F-actin network where they contribute to contractile force generation. These myosin binding dynamics, and the resulting force generation, thus depend on the number of myosin filaments and the density of actin in the gel^35^. Finally, we found that it is essential to account for the known load-dependence of the myosin binding dynamics^36^ (Supplementary Section 3.4 and Supplementary Fig. 7). As the force builds up, the load-dependent myosin kinetics results in an increased number of motors being engaged in force generation.

The inherent positive feedback between the slow build-up of viscoelastic network stresses and the active force generation by fast load-dependent myosin binding kinetics in our model gives rise to the intricate contraction dynamics of the pillar ring, in quantitative agreement with our experiments (Fig. 2i). When possible, the parameters of our dynamic 1D contractility model were chosen on the basis of literature values (Supplementary Table 1). The remaining parameters were fully constrained by fitting the model to a single contraction curve at one pillar stiffness (Fig. 2i). This model accurately predicted the dynamics and steady-state values of the actively generated forces over a broad range of pillar stiffness (steady state: Fig. 2e–g and Extended Data Fig. 6), including the stiffness-dependence of the contraction velocities (Fig. 2j). The stiffness insensitivity of the steady-state force can be explained by our contractility model (Fig. 2g, solid line), provided that the network’s elastic response is much softer than the pillar stiffness and can thus be neglected (Supplementary Section 3.1 and Supplementary Figs. 4 and 5). Conceptually, the load sensitivity of the myosin binding kinetics results in active force generation, which is largely controlled by the internal stress of the actin network and is insensitive to the stiffness of the frame. In contrast, when only accounting for an actin density-dependent contractility as in other models^35^, the stiffness dependence was predicted incorrectly (Fig. 2g, dashed line, Supplementary Section 3.3 and Supplementary Figs. 5 and 6). Finally, our 1D contractility model together with the experimental results could be used to estimate that the generated mechanical power of the exoskeleton in the pillar ring assay peaks was 43 × 10^−18^ W (Supplementary Section 3.5 and Supplementary Table 2).

## Actuating spatial transformations of complex 3D objects

**Fig. 3:**
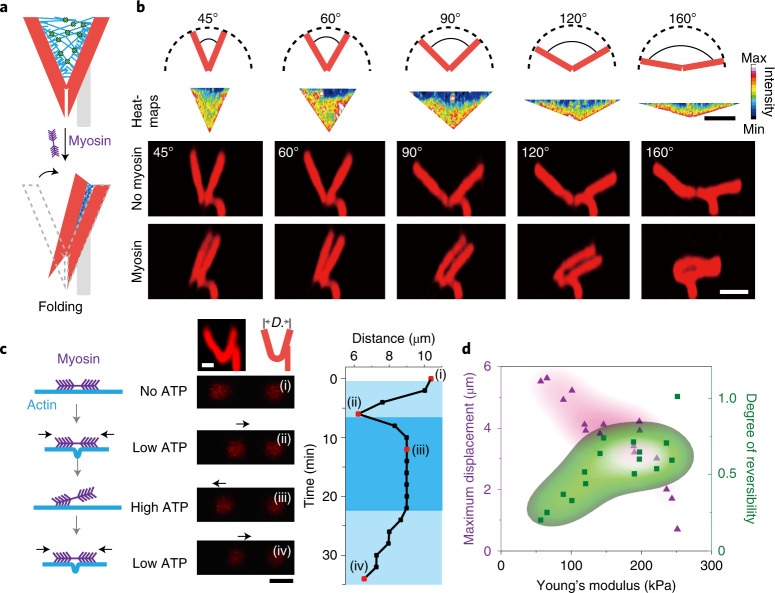
Reversible operation of hinged 3D V units by switching ATP concentrations. **a**,**b**, Schematics of free-standing V units (**a**) and the active folding of V units with different opening angles (0.5 mM ATP; **b**). The heatmaps in **b** show the density of the actin–myosin network on the V units (*n* = 8). Scale bar, 10 µm. The z-projections illustrate the shape morphing of V units before and after triggering with myosin. Scale bars, 10 µm. **c,** Reversibility of the closure of V units with round joints in response to different ATP concentrations: 0 mM (i), 0.5 mM (ii), 4 mM (iii) and 0.5 mM (iv) ATP. Scale bars, 5 µm. The images show the V units viewed from the side (top left) and from above (i-iv). The plot describes the reversible opening and closure of the V units over time. *D* denotes the distance between the two arm tips. **d**, Density maps of maximal displacement (pink) and reversibility (green) versus Young’s modulus. The degree of reversibility was determined from the distance between the reversed and contracted states. The density map is based on data points that were measured in independent experiments. The reconstituted actin and myosin networks are not shown in this figure. Source data

**Extended Data Fig. 8:**
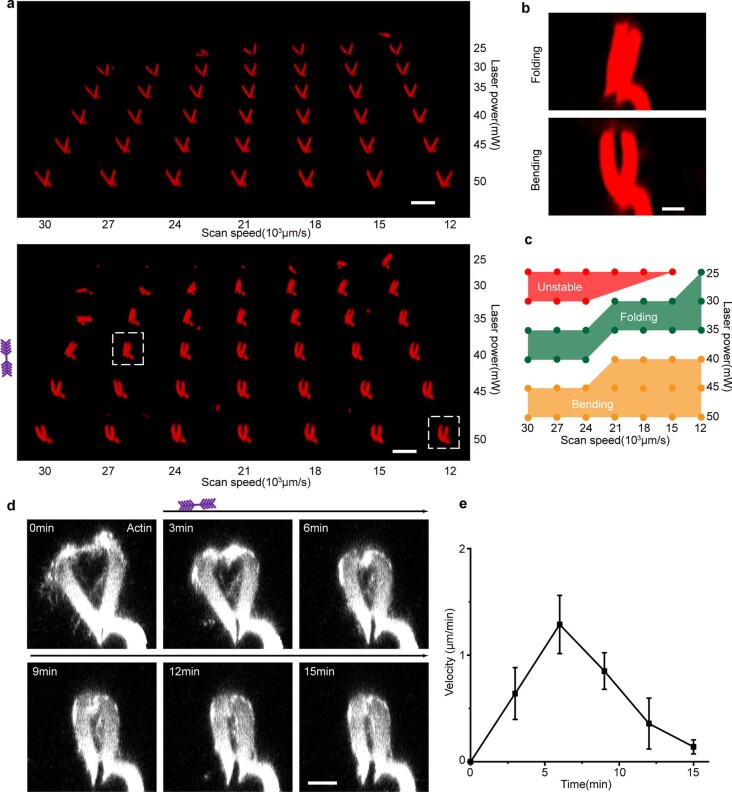
Contraction behaviors of V units depend on the fabrication parameters. **a**.) Printing of V units with different laser powers (25–50 mW) and scan speeds (120000–300000 µm/s). Scale bar, 20 µm. **b**.) Folding and bending of V units after deformation. Scale bar, 5 µm. **c**.) Phase diagram of shape transformation for the V units that was fabricated with different parameters. **d**,**e**.) Contraction dynamics of V-hinge follow the dynamic behavior of pillar rings with an initial acceleration phase and a deceleration phase. d.) Dynamics of V hinge. Scale bar, 10 µm. The images illustrate the side view of the structure. Fluorescence signals in d. are from Alexa Fluor 647 phalloidin labeled actin. e.) The tip velocity of V hinge during contraction. Data points in e. are shown as mean ± s.d (n = 3). The V unit in d. has a similar design as the structures in a., but the printing size is scaled up by a factor of two in all dimensions. Source data

Having gained a conceptual understanding of the force generation and contraction dynamics of the actomyosin exoskeleton on soft frames with simple geometries, we next turned to applications involving complex 3D structures with programmable transformations. The central idea was to assemble 3D structures from modules with tunable stiffness to perform controllable deformations. A key structural element to advance complexity to the next level is a hinge module. Thus, we designed a V-shaped hinge with two stiff arms (pillars) connected by a soft joint (arm/joint thickness ratio = 4:1) and a stabilizing apex to inhibit overstretching to angles beyond 180° (Fig. 3a and Extended Data Fig. 1c). Upon myosin activation, the hinge is actuated, that is, the free arm rotates about the soft joint towards the fixed arm, which follows the dynamic behaviour of pillar rings and exhibits an initial acceleration phase and a deceleration phase (Supplementary Video 2 and Extended Data Fig. 8). The active closure of V units can be accomplished for a range of convex angles, for example, from 45 to 160° (Fig. 3b), and can be tuned by the fabrication parameters (Extended Data Fig. 8).

Further, to explore the possibilities for reversible operation of our protein hydrogel devices, we designed an alternative round-joint V unit with homogeneous elasticity (Supplementary Fig. 2). Because the activity of myosin motors is sensitive to ATP concentration, reversible shape transformation can be induced by switching between high and low ATP conditions (Fig. 3c and Supplementary Video 3). A low concentration of ATP (0.5 mM) can initiate the active closure of the hinge, as demonstrated above. In contrast, high concentrations of ATP (4 mM) cause myofilaments to detach from the actin exoskeleton, resulting in a relaxation of the elastic hydrogel structures. We successfully performed two repeat contractions by manually changing the ATP concentrations (Fig. 3c). After the second iteration, the structures started to lose their reversibility in response to high ATP concentration. Robust reversibility is partially hampered by the non-reversible breakage and crosslinking of the actin networks^26,29^. We also noticed that softer structures showed larger displacements, but lower reversibility, due to greater energy dissipation^37^. Thus, structures with different rigidity exhibit opposite trends between displacement and reversibility (Fig. 3d). To engineer a reversible shape change, we sought an optimal trade-off between these factors.

**Fig. 4:**
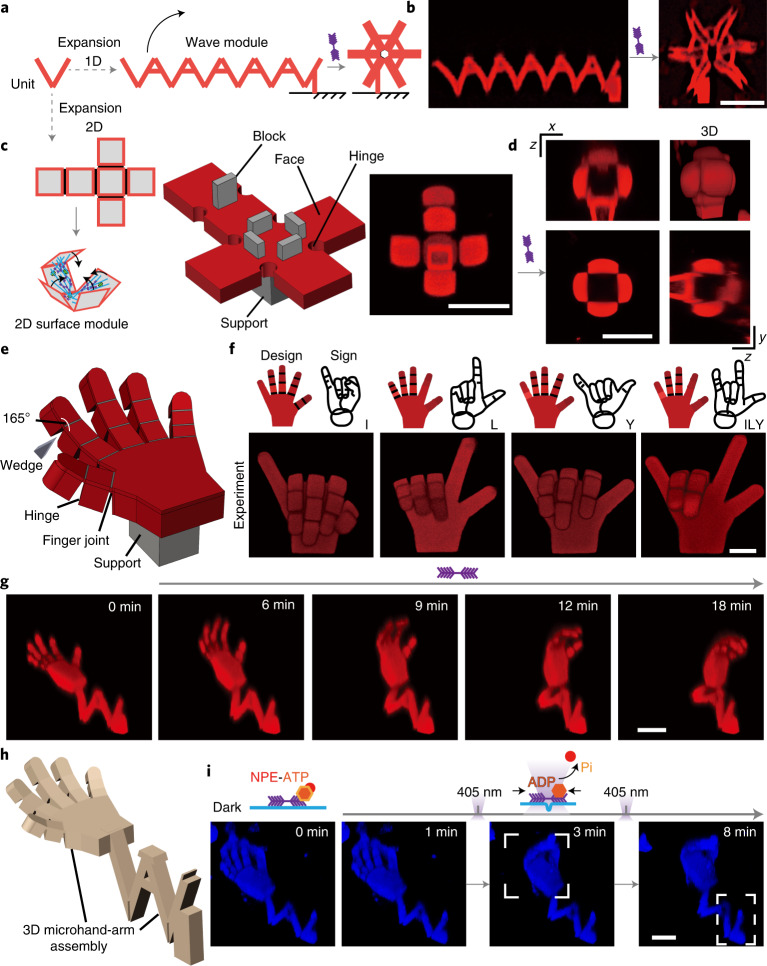
Large-scale transformations of complex 3D structures by targeted biomimetic actuation. **a**,**b**, Schematic design (**a**) and experimental observations (**b**) of concatenated V units demonstrating complex coiling dynamics upon exoskeleton actuation. Scale bar, 20 µm. **c**,**d**, Schematic of a cubic box actively self-folding from a cruciform precursor (**c**) and 3D imaging demonstrating cube closure upon addition of chemical energy (0.5 mM ATP; **d**). Scale bar, 20 µm. **e**,**f**, Design (**e**) and experimental observations (**f**) of microhands displaying different gestures. Scale bar, 10 µm. The gestures are taken from American Sign Language. To program the hand gestures, the hinges were selectively designed with triangular-shaped blocker modules (wedges). These wedges can increase the stiffness of the joint and constrain movement. **g**,**h**, Schematic (**g**) and dynamic actions (**h**) of the robotic arm. Scale bar, 10 µm. **i**, Spatiotemporally induced sequential actuation of a modular robotic arm by light (1 mM NPE-caged ATP). Scale bar, 10 µm. Pi denotes the phosphate generated during ATP hydrolysis. The blue signals in **i** are produced from Alexa Fluor 647 phalloidin-labelled actin. The reconstituted actin–myosin networks are not shown in **a**–**g**. The active contractions in **b**–**g** were initiated with 0.5 mM ATP. 3D views of the structures in **b**–**d**, **f**–**g** and **i** were imaged by confocal microscopy.

**Extended Data Fig. 9:**
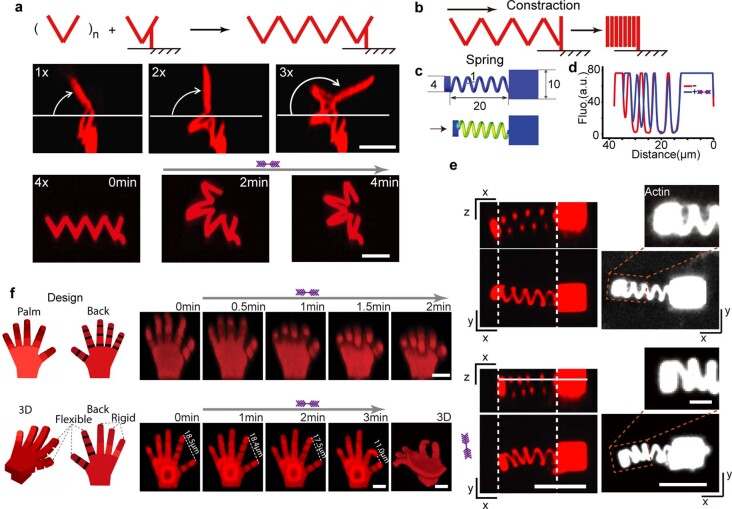
Large-scale transformations of complex 3D structures upon exoskeleton actuation. **a**.) Schematic design and experimental results of V-unit combinations demonstrating complex coiling dynamics under applied active forces. Scale bar, 20 µm. **b**.) Contraction of hydrogel microstructures. **c**.) Schematic design and **d**,**e**.) experimental results of a contractile protein hydrogel spring. Scale bar, 20 µm. Scale bar (zoom), 5 µm. The dimensions in c. are given in micrometers. The line plot in d. represents the spring contraction along its axis before and after trigging (line selection shown in e.). Fluorescence signals of actin networks are from Alexa Fluor 647 phalloidin labeled actin. **f**.) Anthropomorphic gestures of hydrogel microstructures, grasping and ‘OK’. Scale bar, 10 µm. Source data

Scaling up the mechanical work performed by our actomyosin-actuated protein-based robots can now be achieved by concatenating active modules to engineer larger structures. For example, by concatenating the V units, large modular architectures can be assembled, as illustrated in Fig. 4a. Specifically, V units were combined to create a zigzag module, and the connection sites between V units were stabilized to permit folding only on the bottom joints. The actomyosin actuators then independently triggered the closure of all V units, resulting in a rapid curling of the zigzag module (Supplementary Video 4). Instead of using one large hinge angle, 90 and 135° angle folding can be accomplished by combining two or three 45° V units, respectively (Extended Data Fig. 9a). Similarly, large angles that are impossible to achieve with a single unit, such as 180 and 360°, can also be successfully accomplished by concatenating modules. The spring-like zigzag module comprising six V units could revolve around the first joint on the right, its long V chain circularly coiling up and finally forming a closed hexagonal star (Fig. 4b). Besides curling up, a hydrogel spring-shaped structure with additional degrees of freedom can contract along its axis upon myosin-triggered actuation (Extended Data Fig. 9b–e). These examples demonstrate the versatility and potential upscaling of programmable mechanical operations that can be achieved with our protein-based active robotics.

Another desired feature of active biomimetic devices is the ability to fold two-dimensional (2D) surfaces into complex 3D shapes. To accomplish this, we expanded our approach to generate a more intricate shape-morphing, such as the self-folding of a hydrogel cube from a cruciform precursor consisting of six hinged panels (Fig. 4c,d and Extended Data Fig. 1d). The solid-supported central face was connected to the other five free-standing faces through inner hinges. An extra rigid block was placed between the hinging faces to serve as a sill for controlling the folding angle. The flat panels and rigid block formed an angle of 90°, which enabled a simple 2D self-folding sheet to yield a 3D cube under active contraction (Supplementary Video 5).

To further extend the functional complexity of our protein-based robotics, we created programmable and reversible microscale robots with light-induced spatiotemporal control. First, we employed the bioactuated 3D protein hydrogel to mimic grasping microhands, consisting of a panel with five attached fingers (Fig. 4e). The connections between phalanges and the palm were mediated with soft joints. In our design, the actomyosin exoskeleton functions as an external ‘muscle’ layer to actuate the five fingers into a grasping shape with respect to the palm (Extended Data Fig. 9f and Supplementary Video 6). By designing customized 3D protein scaffolds in silico, we can predefine the rigidity and flexibility of the joints selectively using triangular-shaped blocker modules (wedges; see Methods for more details), and thus we can realize anthropomorphic gestures, such as ‘OK’ (Extended Data Fig. 9f). Even more complicated American Sign Language can be executed independently in different designs, such as ‘**I L**ove **Y**ou’ (**ILY**; Fig. 4f).

**Extended Data Fig. 10:**
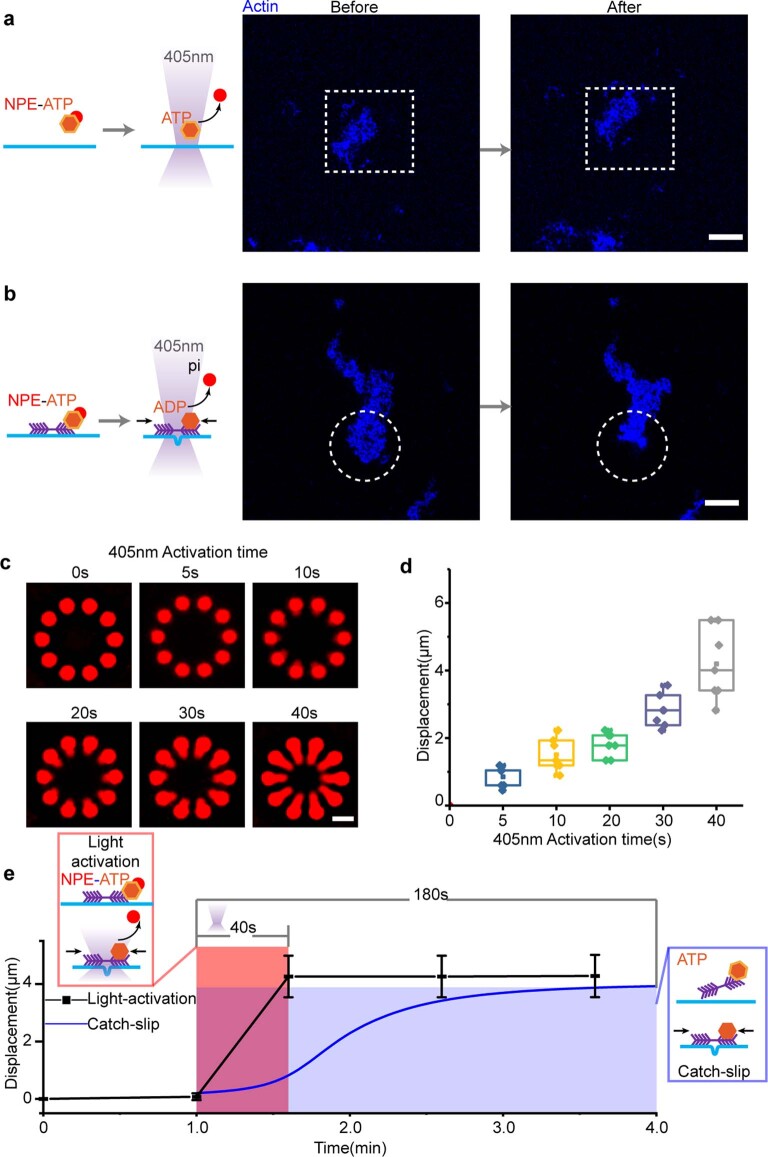
Spatiotemporal control of actin-myosin contraction by UV light. **a**.) Control experiment of photo-activation on actin network without myosin, demonstrating that the UV laser itself will not induce contraction of actin networks. Scale bar, 20 µm. The dashed square represents the activation region. The region was activated with 30 mW 405 UV laser for 2 min. **b**.) Spatially activating actomyosin network contraction. Scale bar, 20 µm. The dashed circle represents the activation region. 405 nm UV laser could precisely activate the local contraction of the actomyosin network. The region was activated with 30 mW 405 UV laser for 24 s. **c**.) Representative imaging (Z Projection) and **d**.) displacement plot (n = 7) of pillar rings activated with different periods of 405 nm UV laser. Scale bar, 5 µm. Lines are median, box limits are quartiles 1 and 3, whiskers are 1.5×interquartile range and points are outliers. Red color in c. represents the signal of hydrogel. Actomyosin networks on the structures are not shown. **e**.) Displacements of pillar ring under light activation. The blue curve represents the simulated active contraction of pillar ring with catch-slip bonds behavior. Data points in e. are shown as mean ± s.d (n = 10). Based on the insights from theoretical modeling, the load dependent binding dynamics of myosin enable a large-scale contraction with a peak velocity on time scales much larger than the molecular dynamics of the motor. This informs us that the response time of the system can be optimized by letting motors bind already before initiating the contraction. In this way the initial slow phase can be avoided and the contraction time can be significantly reduced. Experimentally, when the photoactivation in e. was processed after incubating with myofilaments (1 mM NPE-ATP) for 1.5 h, indeed, the light activation with pre-loaded motors can shorten the response time and speed up the active contractions in comparison with the active contraction that rely on the catch-slip bonds. Source data

Finally, we combined the microhand and the chain of V units to form a miniature robotic arm—fully made from and actuated by proteins (Fig. 4g,h and Supplementary Video 7). Initiating the contraction of the external actomyosin exoskeleton triggered a raising and grasping motion of the arm. As a first proof of concept of reversible operation, the artificial arm was subjected to high-ATP-concentration conditions (4 mM ATP), and responded with a twitch (Supplementary Video 7). We next explored how artificial arm motions can be designed as goal-directed behaviours subject to multistage control. Using photocaged (*P*^3^-[1-(2-nitrophenyl)ethyl] ester (NPE)-caged) ATP as a molecular light sensor, the ATP-dependent actuation module, that is, the actomyosin exoskeleton on the surface of the robotic arm, can be remotely controlled with light (Fig. 4i and Extended Data Fig. 10). Through spatiotemporally targeted release of ATP by illumination with a focused 405 nm diode laser, stepwise arm and hand movements can be guided by light stimuli within 1 min (Fig. 4i and Supplementary Video 8). However, the light activation can only be used for single-run applications due to photodamage. Interestingly, as expected on the basis of our model, the complex structure activated by light and preloaded motors can respond more rapidly than by recruiting motors and energy out of solution, suggesting a new avenue for improving the performance of our system in the future.

## Concluding remarks

To conclude, we have demonstrated the ability to 3D print complex protein-based microrobotics functionalized with a minimal actomyosin exoskeleton as its actuating system. The efficient and scalable operation of reconstituted biological motor assemblies as contractile layers enabled the large-scale, shape-morphing of complex 3D microstructures by converting chemical energy directly into mechanical work. Furthermore, being designed and assembled entirely from biomolecules in a bottom-up fashion, the self-powered soft robotic system constitutes an excellent starting point as a chassis. The performance in terms of speed, force and reversibility may in the future be advanced by operating under automated microfluidics^2^, by optimizing actomyosin network composition^38^ with natural crosslinking proteins^39^ and by integrating other biological or biomimetic modules^11,40^, such as actin recycling systems^41^. We consider this an exciting step in the engineering and programming of arbitrarily shaped biomotor-based actuators for future soft robotics^1^, which no longer rely on the swelling and shrinking of materials (Supplementary Table 3). Within the framework of the bottom-up assembly of life-like systems, as pursued by us and others^1,4,13^, the technology that we have developed here opens new vistas for various applications, such as custom-shaped dynamic sensors and bioassays, microrobots for in vivo biomedical tasks and prototissue engineering, when introduced to the life sciences^42,43^.

## Methods

### Preparation of the BSA solution

First, 3.8 g BSA (Sigma Aldrich), 1 mol% biotinylated-BSA (Thermo Fisher Scientific) and 1.62 ml dimethylsulfoxide (DMSO; 18 v/v%) were added to 20 mM HEPES buffer to prepare a solution with a total volume of 9 ml. The mixture was then centrifuged (20,000 *g*) for 15 min to remove impurities and foam before use. A solution of 85 mM rose bengal (Sigma Aldrich, 330000) was prepared separately. The BSA photoresist (380 g l^–1^) was prepared by mixing BSA resin and rose bengal in a ratio of 9:1 (v/v).

### 3D BSA hydrogel printing

Three-dimensional BSA hydrogel printing was conducted with a Nanoscribe Photonic Professional instrument (Nanoscribe), and 3D structures were designed (Supplementary Fig. 2) and optimized (Supplementary Fig. 8) with Solidworks (Dassault Systèmes SOLIDWORKS Corp.). The printing parameters were defined using Describe (Nanoscribe). The following printing parameters were used (Figs. 2c,d,g,h, 3b,c and 4, Extended Data Figs. 1, 3–5 and 7–10, and Supplementary Videos 2–5, 7 and 8): laser power, 35 mW (70%); scan speed, 30,000 μm s^–1^; slicing distance, 0.3 μm; hatching distance, 0.2 μm. All structures were printed with a ×63 near aperture 1.4 objective in silicone isolator chambers (round, 9 mm diameter, 1 mm depth; Thermo Fisher Scientific), pasted onto round glass coverslips (30 mm diameter, #1.5 thickness; Thermo Fisher Scientific). During printing, the chambers were covered with small coverslips to avoid strong evaporation. After fabrication, the structures were rinsed with phosphate-buffered saline (PBS) buffer (pH 7) to remove excess BSA resin and photoresist. The structures were stored in PBS (pH 7) buffer, which was then further exchanged for other buffer solutions according to the experimental requirements. All experiments, except for scanning electron microscopy (SEM) imaging, were performed in buffer solutions to maintain proper hydration of the protein hydrogel. The structures were biotinylated using BSA doped with BSA-biotin for printing. As a consequence, our structures displayed free biotin groups exposed at the surface.

### Young’s modulus measurement with atomic force microscopy

Atomic force microscopy was performed on a Nanowizard III BioAFM (JPK Instruments) mounted on a Zeiss LSM510 Meta laser scanning confocal microscope (Jena Bioscience). Silicon nitride cantilevers (MikroMasch, XNC12/CR-AU B), with a typical spring constant of 0.32 N m^–1^, were used for force spectroscopy and quantitative imaging. For typical measurements, the set-point force was set to 2 nN, the acquisition speed to 250 µm s^–1^ and the *z* length to 4 µm. The Young’s modulus of microstructures was measured in solutions containing 50 mM KCl, 2 mM MgCl_2_ and 10 mM Tris–HCl (pH 7.5). Data were analysed using JPK data processing software (Version 5.1.4, JPK Instruments). The Young’s modulus was obtained by fitting the extended part of the force–penetration curves (*n* = 900) with a simple Hertz–Sneddon model, considering a quadratic pyramid tip shape and tip angle of 35° (using the JPK data analysis software).

### SEM imaging of hydrogels

To prepare samples for SEM imaging, the BSA microstructures were sequentially exchanged in increasing serial concentrations of acetone (20, 40, 60 and 100%). The samples were then dried with a Leica EM CPD300 automated critical point dryer after immersing in pure acetone. Samples were sputter-coated with platinum–palladium using a high-resolution automatic sputter coater (Cressington, 208HR) at 20 mA under 0.1 mbar argon for 3 × 20 s. The thickness of the applied coatings was measured with a built-in thickness controller to be 2.0 nm. The coated surfaces were viewed using a TESCAN MIRA3 field emission scanning electron microscope operating at an accelerating voltage of 10 kV in scanning electron mode.

### F-actin preparation

Actin filaments were prepared according to a previously published protocol^44^. Briefly, 32 µl rabbit skeletal muscle actin monomers (stock: 2 mg ml^–^^1^, Molecular Probes) and 1.6 µl biotinylated rabbit actin monomers (stock: 10 mg ml^–1^, tebu-bio, Cytoskeleton) were mixed in a 5:1 actin/biotin-actin ratio for a final concentration of 39.6 μM. Polymerization of the mixture (39.6 µM) was induced in F-buffer containing 50 mM KCl, 2 mM MgCl_2_, 1 mM dithiothreitol (DTT), 1 mM ATP and 10 mM Tris–HCl buffer (pH 7.5). The biotinylated actin filaments were labelled and stabilized with 3.96 μM Alexa Fluor 647 phalloidin (stock: 6.6 μM, Molecular Probes) according to the manufacturer’s protocol. An actin-stabilizing solution was prepared by placing 60 μl of 6.6 μM Alexa Fluor 647 phalloidin in a 1.5 ml Eppendorf tube and dried in a vacuum centrifuge at room temperature. The dried powder was dissolved in 5 μl methanol and further diluted with 85 μl labelling buffer containing 10 mM 3-(*N*-morpholino)propanesulfonic acid (MOPS; pH 7.0), 0.1 mM ethylene glycol-bis(β-aminoethyl ether)-*N*,*N*,*N*′,*N*′-tetraacetic acid (EGTA) and 3 mM NaN_3_. Then, the 39.6 μM actin solution was diluted with the labelling buffer to 20 μM actin. Next, 10 μl of 20 μM actin was further diluted with 90 μl actin-stabilizing solution to obtain 2 µM (with respect to the monomers) of Alexa Fluor 647 phalloidin-labelled biotinylated actin filaments.

### Myofilament preparation

Myosin (21 µM stock solution) was purified from rabbit skeletal muscle tissue as previously described^45^. Then, 0.3 µM myofilament assembly (with respect to the monomers) was induced in reaction buffer containing 50 mM KCl, 2 mM MgCl_2_, 1 mM DTT and 10 mM Tris–HCl buffer (pH 7.5). Equilibration of the mixture for approximately 30 min gave us a myofilament median length of 560 nm in our system^26^.

Myosins were labelled with the thiol-reactive dye Alexa Fluor 488 maleimide (Molecular Probes) by a slight variation of the published protocol^26^. In brief, the thiol-reactive dye was dissolved in DMSO to a concentration of 10 mM. The myosin stock was diluted to 2 µM in reaction buffer containing 300 mM KCl, 10 mM Tris–HCl buffer (pH 7.5) and 2 mM MgCl_2_. The solution was deoxygenated for 15 min under vacuum and placed in a N_2_ environment. A 15-fold molar excess (30 µM) of tris(2-carboxyethyl)phosphine (Molecular Probes) was added to the solution and incubated for 1 h at room temperature. Then, a 25-fold molar excess (50 µM) of maleimide dye was added dropwise to the solution with stirring and incubated overnight at 4 °C. The labelled myosin monomers were separated from the remaining dye by gel filtration with a Sephadex G-25 column (GE Healthcare) according to the manufacturer’s protocol using 300 mM KCl and 10 mM Tris–HCl buffer (pH 7.5) as elution buffer. Fractions were measured with an Infinite 200 PRO plate reader (Tecon) and the fraction with the strongest signal was used in experiments. Aliquots were frozen and stored at −80 °C with 50% glycerol; 10% labelled myosin was used for the actin–myosin network imaging.

### Active contraction of 3D printed hydrogel structures

Biotinylated 3D hydrogel structures were transferred from PBS to a buffer containing 50 mM KCl, 2 mM MgCl_2_ and 10 mM Tris–HCl (pH 7.5) by buffer exchange and further incubated with 10 µg ml^–1^ neutravidin (stock: 1 mg ml^–1^) for at least 30 min. Then, free neutravidin was gently washed away with washing buffer containing 50 mM KCl, 2 mM MgCl_2_ and 10 mM Tris–HCl (pH 7.5). The microstructures were further incubated with 0.3 µM (monomer concentration) preformed actin filaments and 2.25 nM neutravidin (neutravidin/biotin-actin, 1:20) for 2 h, followed by careful washing with buffer. For active contraction, 0.5 mM ATP was added to 100 µl preformed myofilaments, and then this solution was further added to the reaction chamber containing 60 µl buffer solution (Figs. 2, 3b and 4a–g, Extended Data Figs. 1–9 and Supplementary Videos 1–7). The imaging was conducted immediately after adding the myofilaments.

Reversibility of the microstructures was achieved by adding 4 mM ATP after the hydrogel structures had been deformed (Fig. 3c,d). The contraction could be recovered by gently washing away the high ATP concentration, and then the buffer was exchanged to the washing buffer with 0.5 mM ATP. All the steps were controlled manually by pipetting, the shear force and dilution effect of which may limit full mechanical reversibility. Therefore, all the washing steps should be performed gently. Because the reversible structure has a round joint (Supplementary Fig. 2c), the distance between the tips of the two arms was used to quantify the dynamics of reversibility.

#### Hand gestures

The 3D microhand gestures (Fig. 4f) were designed with Solidworks according to American Sign Language. The hands (‘OK’, ‘I’, ‘L’, ‘Y’ and ‘ILY’) were independent designs, differing in the rigidity and flexibility of the fingers (the designs are illustrated in Fig. 4f, top, and Extended Data Fig. 9f). To realize these, the computer-aided design (CAD) models of microhands were selectively designed with triangular-shaped blocker modules (wedges) built into the hand during 3D printing. These can increase the stiffness of the joint and constrain movement during active contractions. Therefore, the fingers built and constrained with wedges were non-contractile; only the flexible fingers (without wedges) can perform actions.

#### Photoactivation

Here, 1 mM NPE-caged ATP (adenosine 5′-triphosphate, *P*^3^-[1-(2-nitrophenyl)ethyl] ester (NPE-ATP), Jena Bioscience) was used instead of normal ATP for supplying energy to the preformed myofilaments (Fig. 4i and Extended Data Fig. 10). Actin network-coated microstructures were incubated with 100 µl of 0.3 µM preformed myofilaments and 1 mM NPE-ATP for 0.5–1.5 h, and then imaged in darkness using a LSM 780 confocal microscope. Photocleavage was induced by illumination with a 405 nm laser diode (30 mW). Spatial photoactivation was controlled using the photobleaching mode of the LSM 780 confocal microscope and photoactivation time was controlled by the number of bleaching iterations.

### Myofilament quantification

The numbers of myofilaments in the actin networks were estimated through quantitative fluorescence microscopy. First, 1 µM Alexa Fluor 488 labelled myosin monomers (labelling efficiency 1.3) were diluted to various concentrations (0.001–1 µM). The serial dilutions were performed in protein LoBind Tubes (Eppendorf) and then transferred to poly(L-Lysine)-poly(ethylene glycol) methyl ether (PLL-PEG) passivated microwell plates. The fluorescence intensity (mean) was measured by using the same microscope and camera settings as for the actomyosin gel measurement (Microscope, LSM800; objective, C-Apochromat ×40 and 1.2 W korr; laser power, 1%; detection wavelength, 410–546 nm; gain, 700 V; scaling per pixel: 0.099 × 0.099 µm^2^). For fluorescence intensity measurements, the microscope was focused on the solution far from the chamber surface, where the intensity was maximal, and images were taken in different areas (*n* > 4). The fluorescence signals were plotted against myosin concentration. Then, the average local concentration of the labelled myosin was calculated in accordance with the standard curve. The myofilament number was further calculated according to the labelling ratio (10%), local volume (inner, 1,055 µm^3^; ring, 1,225 µm^3^), average myosin monomer density in myofilaments (560 molecules µm^–1^; ref. ^46^) and the average myofilament length (0.56 µm; ref. ^26^). The calculations were repeated for nine independent experiments.

### Image analysis

Image analysis and processing were carried out with Fiji and ZEN software. To generate the actin network heatmaps, actin networks on pillar rings were imaged in the same experimental session using the same microscope and camera settings. The actin networks were prepared according to the above method and imaged after incubation for 2 h. The *z*-stacks of actin networks were projected with maximum intensity separately. The network patterns from eight replicate experiments were then incorporated into a *z*-stack by importing the *z*-projection images as a sequence. The images in the *z*-stack were further aligned in Fiji with the plugin ‘MultiStackReg’ (transformation: rigid body) and projected (*z*-projection) with average intensity. A 16 colour lookup was chosen for the generation of heatmaps (fluorescence calibration range, minimum to maximum: 0–50,000).

### Statistics and reproducibility

Intensity curves correspond to at least three successfully repeated experiments, including those in Fig. 2c,g,i,j, Extended Data Figs. 3d, 4f,g, 5b,d, 7b–e, 8e and 10d,e, and Supplementary Figs. 1, 5a and 6a. The number of replicated experiments is given in the respective figure captions. The experiments presented in Figs. 2b,d and 3b and Extended Data Figs. 3c and 5c were performed eight times under identical conditions. The independent experiments reported in Extended Data Figs. 5e and 10c were performed six and seven times, respectively. All other experiments were performed three times independently under identical conditions, including those described in Figs. 2e,h and 4b–d,f,g,i and Extended Data Figs. 1, 3a,e–h, 4b–d, 5f, 6, 8a,b,d,e, 9a,e,f and 10a,b. The statistical tests in Fig. 2c and Extended Data Figs. 5b,d were analysed by ANOVA one-way statistical tests (significance level, 0.05; mean comparison, Tukey; tests for equal variance, Levene; power analysis, actual power). All *P* values are given in the respective figure captions.

### Force quantification

Deflection profiles were extracted from the confocal microscopy images of the deformed pillars with Fiji. To this end, the data were first binarized. For a better separation of adjacent pillars, the built-in watershed algorithm was used. The images could then be analysed with the help of Fiji’s particle analysis tool, which tracked the cross area of the pillars and calculated the position of the ‘centre of mass’ for each slice of the pillar. To obtain an estimate of the force, generated by the actomyosin gel, the pillar profile was predicted from elasticity theory (Supplementary Section 1). Subsequently, a one-parameter fit was performed to obtain the force. For the fit, the data from a height below 2 µm were ignored, because the pillar has a stiffer foot, which is hardly deflected. Note that the pillars were not always perfectly straight before they were deformed by the myosin activity. To exclude this effect from the analysis, we corrected the observed final deflection by subtracting the fitted initial profile. The diameters and heights of the pillars were measured by SEM and confocal imaging. The codes for force calculation are available upon request.

The pillar shear strain is defined as Δ*x*/*L*, where Δ*x* is the displacement in the horizontal direction at the tip of the pillar and *L* is the length of the pillar.

### Reporting Summary

Further information on research design is available in the Nature Research Reporting Summary linked to this article.

## Online content

Any methods, additional references, Nature Research reporting summaries, source data, extended data, supplementary information, acknowledgements, peer review information; details of author contributions and competing interests; and statements of data and code availability are available at 10.1038/s41563-022-01258-6.

## Supplementary information

Supplementary Information Supplementary text, Figs. 1–8, Tables 1–3 and the captions for Videos 1–8.

Reporting Summary

Supplementary Video 1 Active contractile dynamics of pillar ring array in response to the motor addition.

Supplementary Video 2 Active force driving free-standing V-unit folding.

Supplementary Video 3 Energy-sensitive reversible V-unit contraction.

Supplementary Video 4 Unit coordination-promoted hydrogel ‘Zigzag’ coiling up.

Supplementary Video 5 Active force actuating self-folding of hydrogel cubes.

Supplementary Video 6 Bioactuated 3D protein hydrogel to mimic grasping microhands.

Supplementary Video 7 Dynamic actions of the robotic arm.

Supplementary Video 8 Sequential and spatiotemporal photoactivation of the artificial arm.

## Source data

Source Data Fig. 2 Statistical source data.

Source Data Fig. 3 Statistical source data.

Source Data Extended Data Fig. 2 Statistical source data.

Source Data Extended Data Fig. 3 Statistical source data.

Source Data Extended Data Fig. 4 Statistical source data.

Source Data Extended Data Fig. 5 Statistical source data.

Source Data Extended Data Fig. 6 Statistical source data.

Source Data Extended Data Fig. 7 Statistical source data.

Source Data Extended Data Fig. 8 Statistical source data.

Source Data Extended Data Fig. 9 Statistical source data.

Source Data Extended Data Fig. 10 Statistical source data.

## Data Availability

All data used in this paper are available at Figshare through the identifier 10.6084/m9.figshare.19345874.v1 or from the corresponding authors upon request. Source data are provided with this paper.

## Code availability

All code used in this paper is available at GitHub (https://github.com/JFlommersfeld/Actomyosin-contractions-in-soft-pillar-rings) or from the corresponding authors upon request.
