# Supplementary material for: 3D printed protein-based robotic structures actuated by molecular motor assemblies
Source: Nat Mater. 2022 May 26;21(6):703–9. doi: 10.1038/s41563-022-01258-6 (PMC9156402; doi:10.1038/s41563-022-01258-6)
Supplement: Supplementary file 1 — Supplementary text, Figs. 1–8, Tables 1–3 and the captions for Videos 1–8. [file 41563_2022_1258_MOESM1_ESM.pdf]

nature portfolio

https://doi.org/10.1038/s41563-022-01258-6

Supplementary information

3D printed protein-based robotic structures actuated by molecular motor assemblies

In the format provided by the authors and unedited

Supplementary information for

3D printed protein-based robotic structures actuated by molecular motor assemblies

Haiyang Jia[1], Johannes Flommersfeld[2,4], Michael Heymann[1,3†], Sven K. Vogel[1,†], Henri G. Franquelim[1], David B. Brückner[2], Hiromune Eto[1], Chase P. Broedersz[2,4*], and Petra Schwille[1*]

[1]Max Planck Institute of Biochemistry, Am Klopferspitz 18, D-82152 Martinsried, Germany.

[2]Arnold Sommerfeld Center for Theoretical Physics and Center for NanoScience, Ludwig-Maximilians-Universität München, Munich, Germany.

[3]Institute of Biomaterials and Biomolecular Systems, University of Stuttgart, Pfaffenwaldring 57, D-70569 Stuttgart, Germany

[4]Department of Physics and Astronomy, Vrije Universiteit Amsterdam, 1081 HV Amsterdam, The Netherlands

[†]These authors contributed equally to this work.

*Correspondence to: schwille@biochem.mpg.de and c.p.broedersz@vu.nl

This supplementary file includes:

Supplementary Text
Supplementary Figures. 1-8
Supplementary Tables 1-3
Captions for Supplementary Videos 1-8

Other Supplementary Materials for this manuscript include the following:

Supplementary Videos 1-8

Supplementary Text

Table of Contents

1. Theoretical pillar profile 2
2. Estimation of the number of force-generating myosin filaments 3
3. 1D contractility model 4
   - 3.1 Significance of the elastic response of the network 4
   - 3.2 A dynamical model for contractile actomyosin in soft frames 5
   - 3.3 The density dependence of the active contractility 9
   - 3.4 The myosin binding and unbinding dynamics 10
   - 3.5 Performed work and power generation 13

1. Theoretical pillar profile

To derive an analytic expression for the expected pillar profile, we assume that (i) the pillar consists of a linear elastic material, characterized by its Young's modulus E, and (ii) the largest contribution to the torque on the pillar stems from forces acting on its tip. The second assumption is justified by observation that the spanning network is predominantly attached at the top of the pillar (Extended Data Fig. 3e). We thus approximate the force applied by the actomyosin gel on the pillar by a point force F_{p} at the tip of the pillar. Since the pillar length (15 µm) is much larger than the pillar width (2 µm), we assume the pillar to be thin compared to its length and the deflection to be small enough such that we can neglect higher-order derivatives of the displacement x with respect to the height z, its shape is described by the following differential equation[1]:

$$\frac{d^2x(z)}{dz^2} = \frac{4F_{\mathrm{p}}}{E\pi r^4}(L - z) \tag{S1}$$

Here, L and r denote the total length and radius of the pillar, respectively. Integrating this equation twice and assuming a fixed boundary at $z = 0$, such that $x(0) = 0$ and $x'(0) = 0$, yields the pillar profile

$$x(z) = \frac{4F_{\mathrm{p}}}{E\pi r^4}\left(\frac{L}{2}z^2 - \frac{z^3}{6}\right). \tag{S2}$$

Here, the force F_{p} is the only free parameter, while the Young's modulus E is measured experimentally (Extended Data Fig. 2) and the pillar dimensions L and r are controlled in the printing process. We therefore perform a one-parameter fit of Eq. (S2) to the experimental pillar profiles, as shown in Fig. 2f.

From Eq. (S2), the displacement of the tip of a pillar is given by

$$\Delta x \equiv x(L) = \frac{4L^3}{3E\pi r^4} F_{\mathrm{p}}. \qquad \text{(S3)}$$

which is equivalent to a stretched spring with an effective spring constant $k_{\mathrm{p}} = 3E\pi r^4/(4L^3)$.

2. Estimation of the number of force-generating myosin filaments

Based on the force measurements that we obtain from fitting Eq. (S3) to the measured pillar profiles, we can estimate the number of myosin filaments that effectively contribute to the pillar deflections and compare it to the total number of myosin filaments in the system. To relate the myosin filament force to the pillar deflection forces, we calculate the active stress at the edge of the gel that would be generated by N_{M} myosin filaments and then relate this to the force per pillar. For this we assume that the actomyosin gel has a disc like geometry (see discussion in Section 3.2 and Supplementary Figure 4).

The active stress σ generated by a density ρ_{M} of individual myosin filaments of size l_{M}, each generating a force F_{M}, is given by[2,3]

$$\sigma = \rho_{\mathrm{M}} F_{\mathrm{M}} l_{\mathrm{M}}. \qquad \text{(S4)}$$

The density of myosin filaments can be expressed through the number of myosin filaments N_{M} and the dimensions of the gel (ring radius D and gel height h) as

$$\rho_{\mathrm{M}} = \frac{N_{\mathrm{M}}}{h\pi\left(\frac{D}{2}\right)^2}. \qquad \text{(S5)}$$

The pillars are spaced by a distance d. To relate the stress in Eq. (S4) to the force per pillar, we assume that the stress over a region of size d focuses on a pillar, which yields the following expression for the force per pillar:

$$F_{\mathrm{p}} = \sigma h d = 4\frac{N_{\mathrm{M}} F_{\mathrm{M}} l_{\mathrm{M}}}{ND} \qquad \text{(S6)}$$

Here, N denotes the number of pillars. The expression in Eq. (S6) allows us to estimate the number of force-generating myosin filaments from the measured pillar forces and the geometry of the pillar rings.

A single skeletal muscle myosin II filament can generate forces in the range $20 - 60$ pN.[4-6] For a ring of diameter $D = 15$ µm with $N = 10$ pillars, myosin filaments of length 0.6 µm and a measured force per pillar of about 130 pN, we thus get an estimate of a 100 – 400 motor filaments that effectively contribute to the pillar force compared to the total number around 8×10^3 myosin filaments in the system as estimated based on measurements of the fluorescence intensity (Supplementary Figure 1).

3. 1D contractility model

To obtain a better understanding of how the motor-induced contractility of the network leads to the observed acceleration effect during the contraction (Fig. 2i), we employ a simple one-dimensional model, to which we can couple the load-dependent myosin binding and unbinding dynamics. This model represents the elastic components of the system, such as the pillars and the elastic response of the network as effective linear springs, while the viscous behavior of the system is represented by a viscous dashpot. The motor activity is then represented by a contractile force applied to the frame.

In general, actomyosin networks can be described as active viscoelastic gels[7,8], but the extent to which the elastic response of the network plays a role depends on the timescale on which the system is observed and experimental factors such as cross-linking. Since the actomyosin network is coupled to elastic frames in our experimental setup, the elastic response of the network will only significantly affect the contraction behavior if the effective spring constant of the network is similar in magnitude or larger than the stiffness of the pillars.

To identify which aspects of the viscoelastic actomyosin gel need to be included in our theoretical description, we first consider the final contraction force as a function of the pillar stiffness in Section 3.1. This analysis reveals that the elastic response of the network can be neglected and we thus proceed with a purely viscous description of the actomyosin gel. We formulate the basic model in Section 3.2 and connect the effective friction coefficient of the dashpot to the rheological properties and the dimensions of the actomyosin gel. We then study different contributions to the time dependence of the contractile force. In Section 3.3, we include the density dependence of the actomyosin contractility in our model and show that this alone is not sufficient to capture the observed contraction behavior. We then refine the model by accounting for the myosin load-dependent binding and unbinding dynamics of myosin filaments in Section 3.4, which yields a simple predictive model for contractile actomyosin networks in soft frames.

3.1 Significance of the elastic response of the network

When analyzing the final contracted state, the viscous response of the network does not have to be accounted for. Hence, only the elastic response of the actomyosin gel contributes. We characterize the elastic response of the network by an effective spring constant k_{am}. Due to the symmetric configuration of our experimental setup, the system can be described by a 1D model with a spring, representing the elastic response of the actomyosin gel, which is attached to two

springs of zero rest length, representing the pillars. This is justified by the discussion in Section 1, where we have seen that the tip displacement of a bending pillar is equivalent to a stretching spring with an effective spring constant k_{p} (Supplementary Figure 4). Finally, the myosin activity induces an active stress in the system, which leads to a contractile active force F_{A} acting on each of the pillars. Thus, the gel's activity leads to a stretching of the two pillar springs by Δx (Fig. 2a and S4). Note that, due to the symmetry of the system, this corresponds to a deformation of the actomyosin spring by $2\Delta x$. At mechanical equilibrium, this model yields the following force balance equation:

$$2k_{\mathrm{p}}\Delta x = -2k_{\mathrm{am}}\Delta x + 2F_{\mathrm{A}}. \tag{S7}$$

Solving this equation for Δx leads to

$$\Delta x = \frac{F_{\mathrm{A}}}{k_{\mathrm{p}}+k_{\mathrm{am}}}. \tag{S8}$$

which was found previously[9-11]. From Eq. (S8), we would expect a dependence of the final contraction force $F_{\mathrm{p}} = k_{\mathrm{p}}\frac{F_{\mathrm{A}}}{k_{\mathrm{p}}+k_{\mathrm{am}}}$ exerted on the pillars, on the pillar stiffness k_{p}, if k_{am} is larger or of a similar order of magnitude as k_{p} (Supplementary Figure 5a), as e.g. observed for living cells[9]. Furthermore, we would also expect a stiffness dependence if the active force F_{A} itself depends on the pillar stiffness. This case is discussed in Section 3.3. Notably, we could not observe such a dependence in our experiments, but instead measured an approximately constant force of 0.13±0.02 nN/pillar (Fig. 2g). The robustness of the final contraction force in the pillar stiffness is captured by this simple model, if the effective spring constant of the network k_{am} is negligible compared to the spring constant of the pillars k_{p} over the entire experimentally considered range. In this 1D model, we expect $k_{\mathrm{am}} \sim \frac{G_{\mathrm{am}}h}{N}$, where N is the number of pillars and the numerical prefactor depends on the Poisson's ratio of the gel, which we do not know. In our case h is around $4-5$ µm, $N = 10$, and we expect G_{am} to be of order a few Pa on the timescale of the contraction[12]. Based on these numbers we do indeed expect $k_{\mathrm{am}} \ll k_{\mathrm{p}}$. Thus, we will proceed by modelling the actomyosin gel as a viscous fluid and neglects its elastic properties.

3.2 A dynamical model for contractile actomyosin in soft frames

Next, we construct a dynamic model for the contractile actomyosin gels in soft frames. To do this, we have to account for viscous contributions. To do so, we use the same geometry as in Section 3.1, but represent the viscous response of the actomyosin gel by a dashpot with an effective friction coefficient γ_{am} (Supplementary Figure 4).

The friction coefficient can be estimated from the 3D geometry of the contractile network, which allows us to relate the effective friction coefficient γ_{am} of the dashpot to the dimensions and the rheological properties of the actomyosin network. Specifically, we approximate the actomyosin gel as a cylindrical linear elastic medium of radius R and height h (Supplementary Figure 4). The height of the gel was estimated from light microscopy images (Supplementary Figure 3), which showed that the gel attached approximately to the upper 30% of the pillars. Based on this geometry we then derived an expression for the effective friction coefficient γ_{am}.

To support our chosen geometry for modelling the contractile gel, we analyzed which part of the network drives the observed isotropic contractions in the experiment. For this, we investigated the experimental dynamics of contracting rings of different diameters D. First, we kept the number of pillars fixed (Fig. 2d and Extended Data Fig. 5c, left). In this case, we observed that the isotropic contraction failed above a threshold for the ring diameter. This is accompanied by the appearance of disconnected regions and holes in the actin network. When varying the ring diameter while keeping the number of pillars fixed, this affected both the center and the periphery of the spanning actin network. To discriminate which part of the network is essential for an isotropic contraction, we disentangle the two effects by adjusting the number of pillars with the ring diameter, such that the distance between neighboring pillars d remains constant (Fig. 2d and Extended Data Fig. 5c, right). Changing the ring size thus only affects the center of the spanning network and not its periphery near the pillars. We observe again a breakdown of the isotropic contraction above a threshold value for the ring diameter together with the appearance of holes in the spanning actin network. These results indicate that the spanning network in the middle of the pillar ring drives the contraction of the system. We therefore focus on that part of the system for our theoretical modelling.

Based on the approximation of the actomyosin gel as a cylindrical linear continuous medium, we next derive a relation between the pillar force F_{p} and the velocity of the fluid's outer surface. This then allows us to model the network's passive response as a simple one-dimensional dashpot with an effective friction coefficient γ_{am}. To derive γ_{am}, we first consider the viscous stress tensor[13]:

$$\sigma'_{\alpha\beta} = 2\eta\left(\dot{u}_{\alpha\beta} - \tfrac{1}{3}\delta_{\alpha\beta}\dot{u}_{\gamma\gamma}\right) + \zeta\delta_{\alpha\beta}\dot{u}_{\gamma\gamma} \quad \text{(S9)}$$

Here, η denotes the shear viscosity, ζ is the volume viscosity, and the dots denote a temporal derivative. In the absence of external forces and neglecting inertial effects , we then obtain the following differential equation for the strain rate $\dot{u}_{\alpha\beta}$ in an isotropic viscous fluid[13]:

$$0 = \left(\eta + \frac{1}{3}\zeta\right)\nabla(\nabla \cdot \dot{\boldsymbol{u}}) - \zeta\nabla \times (\nabla \times \dot{\boldsymbol{u}}). \tag{S10}$$

Since the load ω is applied radially, $\dot{u}_\varphi$ and all angular derivatives vanish. Additionally, we assume that the fluid remains in a perfectly cylindrical shape. Hence, $\partial_z \dot{u}_r$ and $\partial_r \dot{u}_z$ vanish. We can thus simplify this expression to

$$0 = \left(\eta + \frac{1}{3}\zeta\right)\nabla(\nabla \cdot \dot{\boldsymbol{u}}). \tag{S11}$$

Writing Eq. (S11) explicitly for the different non-zero components in cylindrical coordinates yields

$$0 = \partial_r \left[\frac{1}{r}\frac{\partial(r\dot{u}_r)}{\partial r}\right], \qquad 0 = \partial_z^2 \dot{u}_z \tag{S12}$$

We place the center of the coordinate system in the center of the cylinder. Hence, we know that the velocity at the origin has to vanish due to symmetry, providing the following two boundary conditions: $\dot{u}_r(r = 0) = 0$ and $\dot{u}_z(z = 0) = 0$. Integrating the two equations (Eq. (S12)) with these boundary conditions gives the following velocity field:

$$\dot{u}_r(r) = a_r r, \tag{S13}$$

$$\dot{u}_z(z) = a_z z \tag{S14}$$

To fix the remaining two integration constants a_r and a_z, we consider the stress in the system. We can relate the components of the velocity field to the viscous stress tensor via the constitutive relation:

$$\sigma'_{\alpha\beta} = \eta\left(\partial_\alpha \dot{u}_\beta + \partial_\beta \dot{u}_\alpha - \frac{2}{3}\delta_{\alpha\beta}\partial_\gamma \dot{u}_\gamma\right) + \zeta\delta_{\alpha\beta}\partial_\gamma \dot{u}_\gamma \tag{S15}$$

Here, $\delta_{\alpha\beta}$ denotes the Kronecker delta and repeated indices are summed over. The non-zero components are thus given by

$$\sigma'_{rr}(r) = 2\left(\frac{\eta}{3} + \zeta\right)a_r + \left(\zeta - \frac{2}{3}\eta\right)a_z, \tag{S16}$$

$$\sigma'_{\varphi\varphi}(r) = 2\left(\frac{\eta}{3} + \zeta\right)a_r + \left(\zeta - \frac{2}{3}\eta\right)a_z, \tag{S17}$$

$$\sigma'_{zz}(r) = 2\left(\zeta - \frac{2}{3}\eta\right)a_r + \left(\zeta + \frac{4}{3}\eta\right)a_z. \tag{S18}$$

To derive this, we used that $\dot{u}_r(r = 0) = 0$ and $\dot{u}_z(z = 0) = 0$ due to symmetry. We model the forces applied to the network by the pillars by a continuous load $\omega = NF_\mathrm{p}/A$ on the gel's outer surface $A = 2\pi Rh$ (Supplementary Figure 4), where N denotes the number of pillars per ring. By using these boundary conditions ($\sigma'_{rr}(r = R) = \omega = NF_\mathrm{p}/A$ and $\sigma'_{zz}(z = \pm h/2) = 0$), we can derive the expression for $F_\mathrm{p}(t)$:

$$F_{\mathrm{p}}(t) = \frac{A}{NR}\frac{6\eta}{1+\frac{4\eta}{3\zeta}}\dot{u}_r(r = R). \tag{S19}$$

Note that this expression can be mapped onto the expression one would obtain for an incompressible fluid with an effective viscosity $\eta_{\mathrm{am}}^{\mathrm{eff}} = 6\eta/(1 + 4\eta/(3\zeta))$. We can then simplify this expression to

$$F_{\mathrm{p}}(t) = \eta_{\mathrm{am}}^{\mathrm{eff}}\frac{6A}{NR}v_{\mathrm{p}}(t). \tag{S20}$$

Here, η_{am} denotes the viscosity of the actomyosin gel, $v_{\mathrm{p}} = d(\Delta x)/dt$ is the velocity of the tip of the pillars. This allows us to define an effective friction coefficient γ_{am} as

$$\gamma_{\mathrm{am}} = \eta_{\mathrm{am}}^{\mathrm{eff}}\frac{6A}{NR} = \frac{12}{N}\eta_{\mathrm{am}}^{\mathrm{eff}}\pi h, \tag{S21}$$

where we used $A = 2\pi Rh$.

Using this expression for the effective friction coefficient of the actomyosin network, the force balance condition reads:

$$2k_{\mathrm{p}}\Delta x = 2F_{\mathrm{A}} - 2\gamma_{\mathrm{am}}\frac{d\Delta x}{dt}. \tag{S22}$$

Using $F_{\mathrm{p}}(t) = k_{\mathrm{p}}\Delta x$, this can be rewritten as[8]

$$\frac{dF_{\mathrm{p}}(t)}{dt} + \frac{F_{\mathrm{p}}(t)}{\tau} = \frac{F_{\mathrm{A}}}{\tau}. \tag{S23}$$

where $\tau = \gamma_{\mathrm{am}}/k_{\mathrm{p}}$. For a constant value of F_{A} and an initial force $F_{\mathrm{p}}(t = 0) = 0$, the solution of Eq. (S23) is given by

$$F_{\mathrm{p}}(t) = F_{\mathrm{A}}(1 - e^{-t/\tau}), \tag{S24}$$

implying the following expressions for the displacement Δx and the pillar velocity v_{p}:

$$\Delta x(t) = \frac{F_{\mathrm{A}}}{k_{\mathrm{p}}}(1 - e^{-t/\tau}) \tag{S25}$$

and

$$v_{\mathrm{p}}(t) = \frac{F_{\mathrm{A}}}{k_{\mathrm{p}}\tau}e^{-t/\tau}. \tag{S26}$$

Importantly, however, these predictions quantitatively and qualitatively fail to capture the experimental data (Fig. 2i): Eq. (S25) does not have an inflection point and the velocity in Eq. (S26) correspondingly exhibits no peak at $t > 0$ (Supplementary Figure 5b). This implies that the active force F_{A} cannot be assumed to be constant in this model, but instead needs to increase over the course of the contraction. To account for this, we consider two possible contributions: a density-dependence of the contractility (Section 3.3), and the binding dynamics of the myosin motors (Section 3.4).

3.3 The density dependence of the active contractility

The first effect that could introduce a time dependence of the contractility of the network is the change in network density throughout the contraction process[14-16]. To generate a contractile force, a myosin filament has to connect two distinct actin filaments. The probability that a bound motor will indeed be in such a contractile configuration grows with increasing network density due to the increased number of possible binding sites within reach of the motor. Hence, for lower densities, we expect the active force generated by the actomyosin gel onto the pillars to scale linearly with the actin density and the number of bound myosin filaments. At high densities, steric interactions will counteract the contractile force, to leading order yielding a term that scales quadratically with the actin density. In total, this gives us the following expression for the active force as a function of the actin density[16]:

$$F_{\mathrm{A}}(t) = \chi N_{\mathrm{M}} \rho_a(t)(\rho_a^{\max} - \rho_a(t)) \tag{S27}$$

Here, χ is a phenomenological coupling parameter, N_{M} is the number of bound myosin filaments and $\rho_a^{\max}$ is the maximal possible density that the gel can reach. While it is known that cortex thickness correlates with cortex contractility *in vivo*[17], due to the large areal changes of the considered network in the x-y-plane, for simplicity we considered only the increase in actin density from the in-plane contraction of the network. Furthermore, we neglect the effect of filament alignment. This is justified by the fact that we apply this model to crosslinked networks, in which the effects of motor induced fiber alignment are reduced in comparison to non-crosslinked networks[18].

Inserting the density dependent active force (Eq. (S27)) into Equation (S23) yields

$$\frac{dF_{\mathrm{p}}(t)}{dt} + \frac{F_{\mathrm{p}}(t)}{\tau} = \frac{\chi}{\tau} N_{\mathrm{M}} \rho_a(t)\big(\rho_a^{\max} - \rho_a(t)\big). \tag{S28}$$

Note that the central aspect of the density dependence of the active force is its non-monotonic dependence on ρ_a . This can also be achieved through a different scaling than the one used here. However, we find that our conclusions do not qualitatively depend on the exact scaling of the active force.

Solving Equation (S28) can indeed reproduce the acceleration in the contraction (Supplementary Figure 6a). However, this model lacks predictive power. We demonstrate this by fitting the model to the displacement curve at a single pillar stiffness ($k_{\mathrm{p}} = 35$ pN/µm) that is shown in Supplementary Figure 6a and then try to predict the final contraction force at different pillar stiffnesses. In contrast to the experimental data, this model predicts a strong stiffness dependence of the final force (Fig. 2g, dashed line). Also, the acceleration can only be observed for a

very limited range of pillar stiffnesses (below $k_{\mathrm{p}} = 60\ \mathrm{pN}/\mu\mathrm{m}$). At higher stiffnesses we find a monotonic decrease in velocity (Supplementary Figure 6b) reminiscent of the model with a constant active force (Supplementary Figure 5b). We thus conclude that the density dependence of the active force alone is not sufficient to explain the observed contraction behavior.

3.4 The myosin binding and unbinding dynamics

Another important aspect that we should include into our contractility model is the binding and unbinding of myosin filaments. The filaments can constantly bind to and unbind from the network. In the beginning of the experiment, myosin filaments are in solution and first have to bind to the network to generate contractile forces. Since the active force in Eq. (S27) is assumed to be proportional to the number of bound myosin filaments, the myosin binding kinetics will directly contribute to the time dependence of the network contractility.

To account for this, we extend the model by an equation describing the dynamics of bound myosin filaments:

$$\frac{dN_{\mathrm{M}}(t)}{dt} = k_{\mathrm{on}}[N_{\mathrm{M}}^{\mathrm{max}} - N_{\mathrm{M}}(t)] - k_{\mathrm{off}}(t)N_{\mathrm{M}}(t). \tag{S29}$$

Here, k_{on} and k_{off} denote the binding and unbinding rates of myosin filaments, which are determined by the binding and unbinding rates $k_{\mathrm{on}}^{\mathrm{head}}$ and $k_{\mathrm{off}}^{\mathrm{head}}$ of the individual heads, N_{M} is the number of bound myosin filaments and $N_{\mathrm{M}}^{\mathrm{max}}$ denotes the maximal number of myosin filaments that can contribute to the contraction.

Crucially, the bond lifetime of myosin heads depends on the mechanical load that they experience. In particular, myosin forms so-called catch-slip-bonds with actin[19], meaning that the bond lifetime initially increases with load before it decreases at higher loads. We model this by simplifying the complex myosin binding and unbinding cycle (Supplementary Figure 7a) to a simple binding and unbinding cycle (S7b) with a load-independent binding rate and a load-dependent unbinding rate (Supplementary Figure 7c). For the load-dependent unbinding rate, we use the following phenomenological expression for the unbinding rate of myosin heads as a function of the load per head F_h:[19]

$$k_{\mathrm{off}}^{\mathrm{head}}(F_h) = k_{\mathrm{off}}^{\mathrm{catch}} e^{-\frac{F_h x_{\mathrm{catch}}}{k_{\mathrm{B}}T}} + k_{\mathrm{off}}^{\mathrm{slip}} e^{\frac{F_h x_{\mathrm{slip}}}{k_{\mathrm{B}}T}} \tag{S30}$$

The longer lifetime of the individual heads under load also translates to a longer lifetime of the motor filament as a whole, which then is also expected to effectively behaves as a catch-slip-

bond. This introduces a coupling between the number of bound motor filaments and the stresses that are generated in the system.

To account for this coupling, we start by connecting the binding and unbinding rates of individual myosin heads to the unbinding rates of motor filaments as a cluster of parallel bonds, over which the motor load is distributed equally. The lifetime of individual bonds is assumed to be independent of the state of neighboring heads. The dynamics of the number of bound heads is then equivalent to a biased one-dimensional random walk with forward rate $k_{\text{on}}^{\text{head}}$ and a load-dependent backward rate $k_{\text{off}}^{\text{head}}(F_h)$. A motor filament unbinds in this model when the number of bound heads is zero. This allows us to derive the average unbinding time of a motor filament with initially m bound heads as the mean first passage time $\tau_{m,0}$ of a random walker starting at site m to reach site 0. The average unbinding time of a motor with m bound heads is thus given by[20,21]

$$\tau_{m,0} = \sum_{n=1}^{m} \sum_{i=n}^{N_h} \frac{1}{i \times k_{\text{off}}^{\text{head}}(F_{\text{M}}/i)} \prod_{j=n}^{i-1} \frac{(N_h - j)k_{\text{on}}^{\text{head}}}{j \times k_{\text{off}}^{\text{head}}(F_{\text{M}}/j)}, \tag{S31}$$

where F_{M} denotes the load that a myosin filament experiences in total and N_h denotes the number of heads per myosin filament that interact with an actin filament. Since we assume that every motor filament feels the same average load, we average over all initial conditions to obtain the average unbinding time at every point in time. To do so, we use the steady-state probability distribution $p_i^s(F_{\text{M}})$, which denotes the probability for a motor under load F_{M} to be bound with i heads to an actin filament. Since the myosin heads are stiff compared to the rest of the system (motor elasticity of about 2.5×10^3 pN/µm[18] compared to a typical pillar stiffness of about $100\text{pN}/\mu\text{m}$), this steady state should be reached fast compared to the other timescales in the system and this approximation should be justified for our purposes. The steady-state probability distribution is given by[20]:

$$p_i^s(F_{\text{M}}) = \frac{\prod_{n=0}^{i-1} \frac{(N_h - n)k_{\text{on}}^{\text{head}}}{(n+1) \times k_{\text{off}}^{\text{head}}(F_{\text{M}}/(n+1))}}{1 + \sum_{k=1}^{N_h} \prod_{j=0}^{k-1} \frac{(N_h - j)k_{\text{on}}^{\text{head}}}{(j+1) \times k_{\text{off}}^{\text{head}}(F_{\text{M}}/(j+1))}} \tag{S32}$$

Finally, the myosin filament unbinding rate is

$$k_{\text{off}}(F_{\text{M}}(t)) = \left(\sum_{m=1}^{N_h} p_m^s(F_{\text{M}}(t)) \tau_{m,0} p_m^s(F_{\text{M}}(t)) \right)^{-1} \tag{S33}$$

To complete the model, we need to connect the load on the myosin filaments F_{M} to the force that is exerted onto the pillars. We do this by assuming that the force, generated by the deflected pillars is distributed over all myosin filaments that contribute to the active contraction. Thus, we take a mean-field approach and assume that every motor feels a load

$$F_{\mathrm{M}} = aF_{\mathrm{p}}. \tag{S34}$$

Here, we introduced a phenomenological proportionality constant a. Our contractility model is then defined by the two coupled differential equations:

$$\frac{dF_{\mathrm{p}}(t)}{dt} + \frac{F_{\mathrm{p}}(t)}{\tau} = \frac{\chi}{\tau} N_{\mathrm{M}}(t)\rho_a(t)\left(\rho_a^{\max} - \rho_a(t)\right) \tag{S35}$$

$$\frac{dN_{\mathrm{M}}(t)}{dt} = k_{\mathrm{on}} \times [N_{\mathrm{M}}^{\max} - N_{\mathrm{M}}(t)] - k_{\mathrm{off}}\left(aF_{\mathrm{p}}(t)\right)N_{\mathrm{M}}(t), \tag{S36}$$

Where k_{off} is defined by Equations (S30) – (S33). The parameters related to the (un-)binding dynamics of individual myosin heads $k_{\mathrm{on}}^{\mathrm{head}}$, $k_{\mathrm{off}}^{\mathrm{catch}}$, $k_{\mathrm{off}}^{\mathrm{slip}}$ such as x_{catch} and x_{slip} have been experimentally determined. All of these parameter values can be fixed from the literature at roughly physiological conditions (Supplementary Table 1). This leaves us with seven fit parameters N_h, a, $N_{\mathrm{M}}^{\max}$, $\rho_{\mathrm{a}}^{\max}$, k_{on}, χ and γ_{am}. These parameters were fixed by fitting the model to a single contraction curve at $k_{\mathrm{p}} = 35$ pN/μm (Fig. 2i).

To check that the fitted parameter values take physically reasonable values, we estimate the load that a typical myosin filament will experience in the network in the final contracted state. The final force reached in the contraction in Fig. 2i is 130 pN. From Eq. (S34) and with $a = 0.4$ (Supplementary Table 1) we can thus estimate the load that a typical myosin filament will experience a load of about 50 pN after the contraction reached its steady state. This is in agreement with the forces that individual myosin filaments can produce (see Section 2)[6], demonstrating that the filament loads are within the expected force range for the chosen set of parameter values. Furthermore, the fitted value for the number of heads $N_h = 28$ agrees well with the estimated number of about 30 heads per myosin filament that can interact with an actin filament[2].

To test the predictive power of our contractility model with fully constrained parameters, we used it to predict the stiffness dependence of the contraction in the pillar ring assay. First, we calculated the final contraction force as a function of k_{p}. Our model correctly predicts the low sensitivity of the final force to the pillar stiffness over a wide range, as shown in Fig. 2g (Solid line). Subsequently, we used our model to make predictions for the dynamical properties of the

contraction. In particular, we calculated the maximal velocity that was reached during the contraction. In contrast to the final force, here we observed a strong dependence on the pillar stiffness, which agrees with the experimental observations (Fig. 2j). When accounting for the discreteness of the experimental data in our velocity calculation, we obtain a good quantitative agreement between theoretical prediction and experimental measurements. While for the steady state, the density dependent effects seem to only have a small impact, we find that the density dependence is essential in our model to understand stiffness dependence of the contraction velocity. This is the case since motor filament binding kinetics only couple to the pillar force but not to their displacement and thus not to the pillar stiffness. The density dependence of the contractility in contrast couples the contractility also to the pillar stiffness. We thus conclude that accounting for both the density dependence of the contractility and the (un-)binding dynamics of the myosin filaments is essential for the understanding of the contraction behavior of the fabricated structures.

3.5 Performed work and power generation

We can use our contractility model to estimate the produced work and power during the contraction of our pillar ring assay. Part of the work performed goes into the elastic deformation of the pillar frames and part of the work is dissipated. The dissipated work W_{dis} in the model, however, only accounts for viscous dissipation and does not account for effects of the motors that cannot contribute to the overall contraction. It can thus only serve as a lower bound for the total amount of dissipated energy.

If the pillars are deflected by Δx_{f} in the final contracted state, the total work is given by:

$$W_{\mathrm{tot}} = W_{\mathrm{trans}} + W_{\mathrm{dis}} = N \int_0^{\Delta x_{\mathrm{f}}} k_{\mathrm{p}} \Delta x \, d\Delta x + N \int_0^{\Delta x_{\mathrm{f}}} 2\gamma_{\mathrm{am}} v_{\mathrm{p}} d\Delta x \qquad \text{(S37)}$$

Here, the number of pillars N accounts for the fact that our model is one dimensional. Note that the additional factor of two in the expression for W_{dis} is a consequence of the geometry, which implies that the contraction velocity of the gel is twice the deflection velocity of the pillars v_{p}.

Rewriting the right-hand side of Eq. (S37) as integrals over time yields the following expressions for the dissipative and transmitted components of the generated power:

$$P_{\mathrm{dis}}(t) = 2N\gamma_{\mathrm{am}} v_{\mathrm{p}}(t), \qquad \text{(S38)}$$

$$P_{\mathrm{trans}}(t) = 2Nk_{\mathrm{p}} \Delta x(t) v_{\mathrm{p}}(t). \qquad \text{(S39)}$$

As shown in Supplementary Figure 7d, we observe a strongly peaked profile in both the dissipated and the transmitted power, which is a consequence of the acceleration in the contraction process.

Supplementary Figures

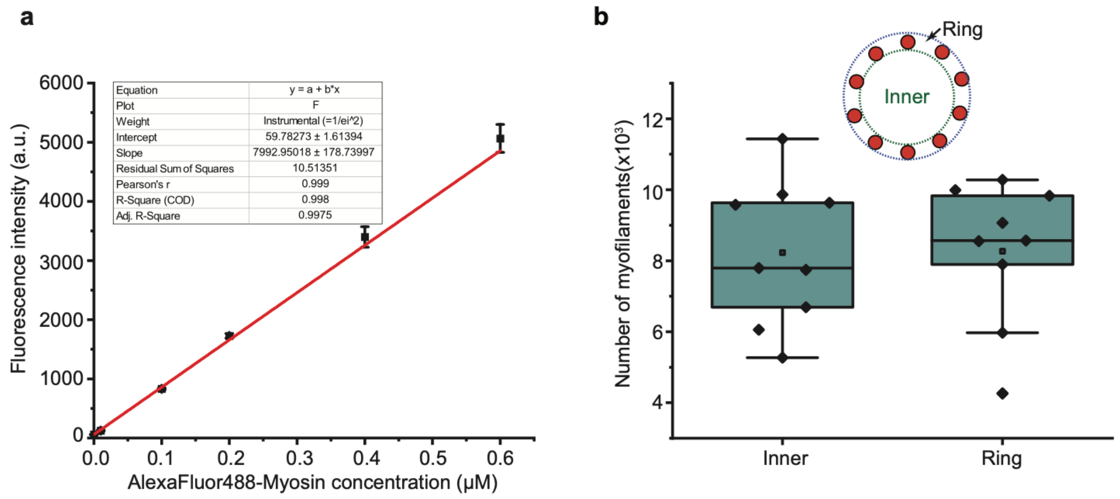

Supplementary Figure 1. a.) Fluorescence intensity calibration of AlexaFluor-488 labeled myosin monomers. Data are shown as mean ± s.d.; n = 3. Fluorescence intensity was measured by confocal microscope with different concentrations of AlexaFluor-488 labeled myosin monomers in solution. b.) The numbers of myofilaments in the actin network on pillar rings (n=8). Box plots in c and f: lines are median, box limits are quartiles 1 and 3, whiskers are 1.5×interquartile range and points are outliers.

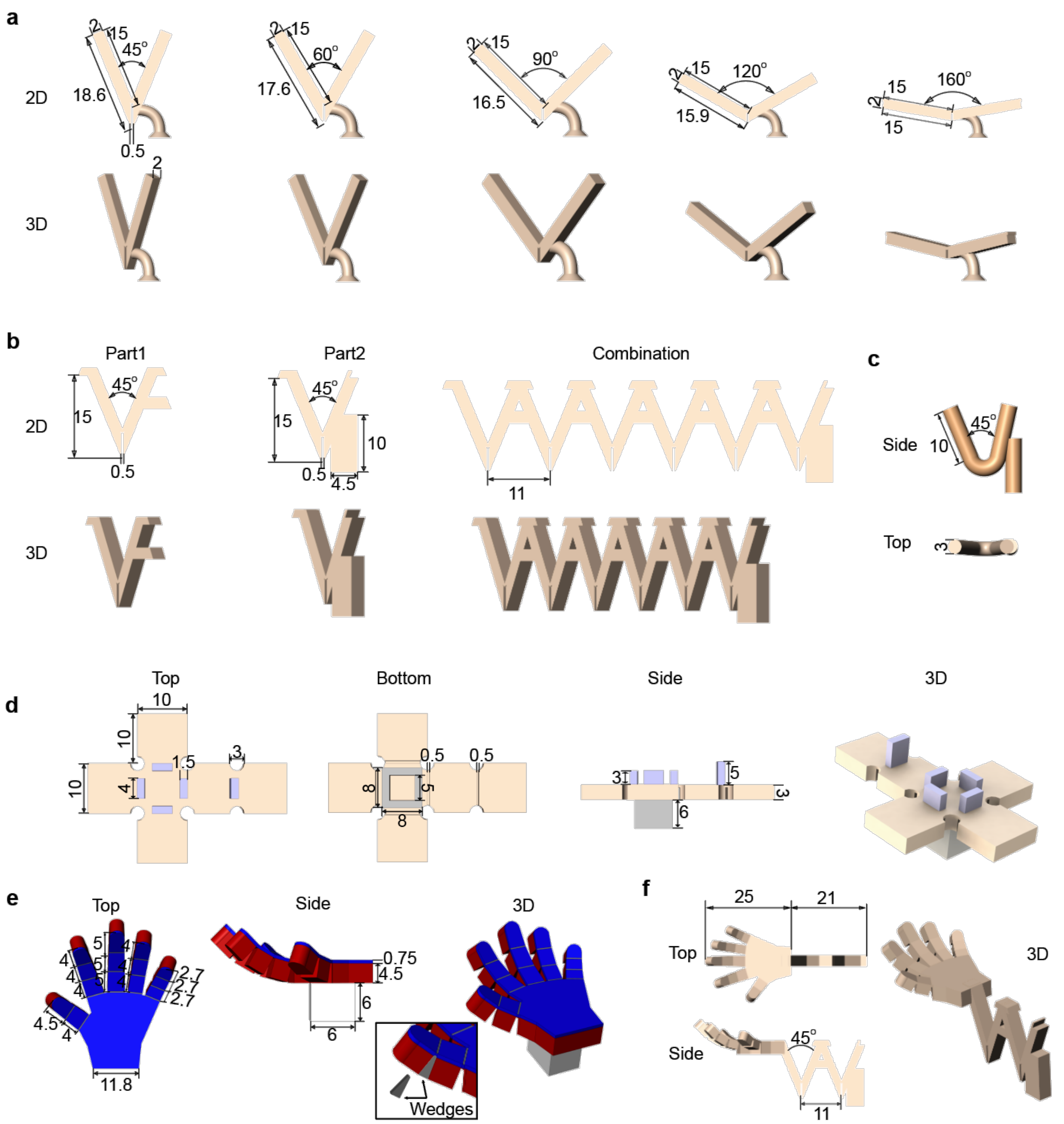

Supplementary Figure 2. Schematic designs and dimensions of the 3D microstructures. a.) V units with different angles in Fig. 3b. b.) Assembly V units for the large-scale combination in Fig. 4a-b. c.) 45° V unit with a round joint in Fig. 3c-d. d.) self-folding cube in Fig. 4c-d. e-f.) Microhands and arms in Fig. 4. To program the hand gestures, the hinges are selectively designed with triangular-shaped blocker modules (wedges). The dimensions in this figure are given in micrometers.

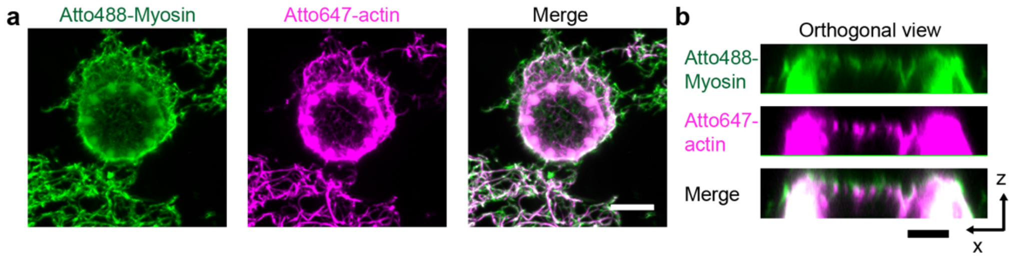

Supplementary Figure 3 a.) Representative fluorescence images of actin-myosin network on pillar rings, projection of z stacks. Actin network was crosslinked with 2.25nM neutravidin. 10 % Atto488-myosin was used for the imaging. Scale bar, 10 µm. b.) Orthogonal views of the actin network in a. The top one-third of the structure was demonstrated, indicating actomyosin network mainly formed on the tip of the pillar ring. Scale bar, 5 µm.

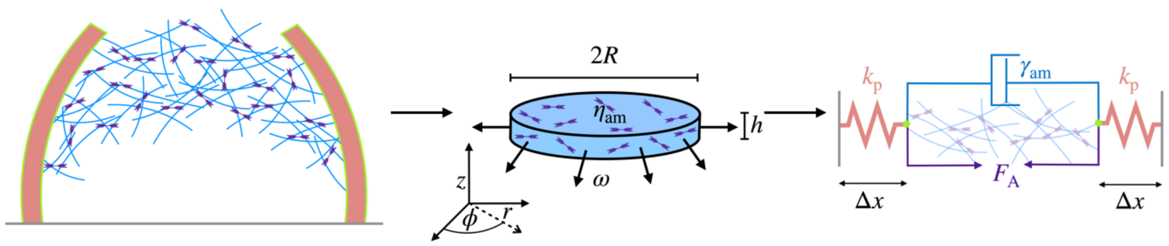

Supplementary Figure 4. Schematic illustration of the mechanical model and the relation between the effective friction coefficient γ_{am} of the dashpot and the material properties and dimensions of the actin gel.

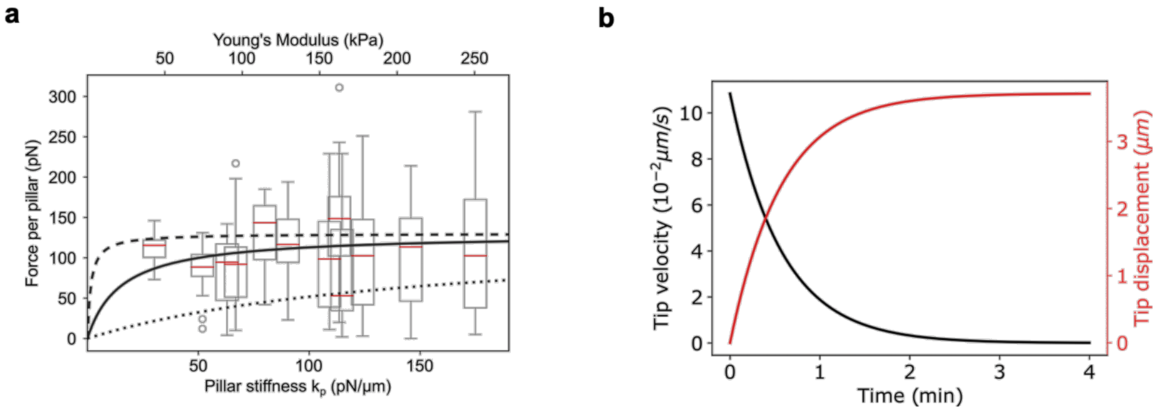

Supplementary Figure 5. a.) Expected force per pillar against pillar stiffness from our one-dimensional contractility model for different values of the actomyosin's shear modulus G_{am} (dashed line: 0.1 Pa, solid line: 1.0 Pa, dotted line: 10 Pa). The box chart represents the experimental measurements of active force per pillar exerted on the pillar ring with different stiffnesses (n=10). Box plots in a: lines are median, box limits are quartiles 1 and 3, whiskers are 1.5×interquartile range and points are outliers. b.) Theoretical contraction dynamics of with a constant active force.

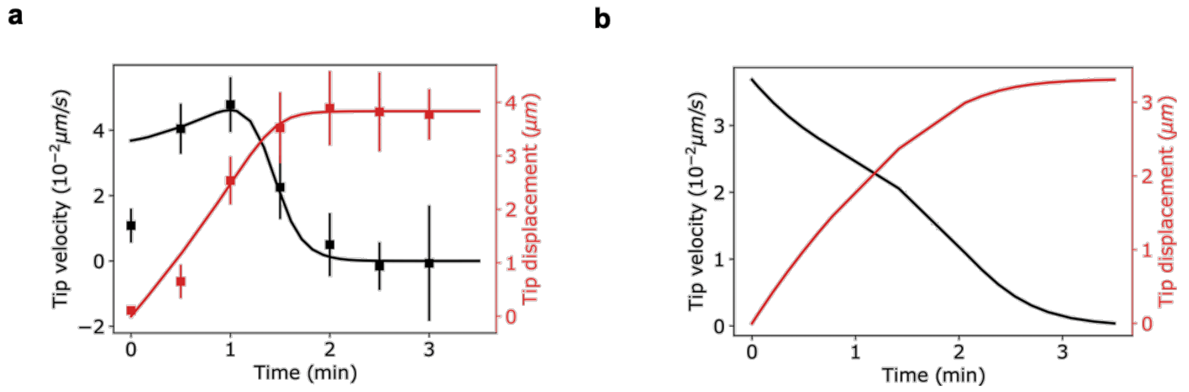

Supplementary Figure 6. Contraction dynamics of the purely density dependent model. a.) Fit to the experimentally observed contraction dynamics ($k_\mathrm{p} = 35$ pN/μm). Data are shown as mean ± s.d.; n = 10 pillars analyzed for each data point. b.) Predicted contraction dynamics at higher stiffness ($k_\mathrm{p} = 100$ pN/μm). Notably, there is no acceleration observable anymore at higher stiffnesses. Fit parameter: $\chi N_\mathrm{M} \rho_a (t = 0)^2 = 50$ pN/s, $\rho_a^\mathrm{max}/\rho_a(t = 0) = 5.4$, $\eta_\mathrm{am}^\mathrm{eff} = 400$ Pas.

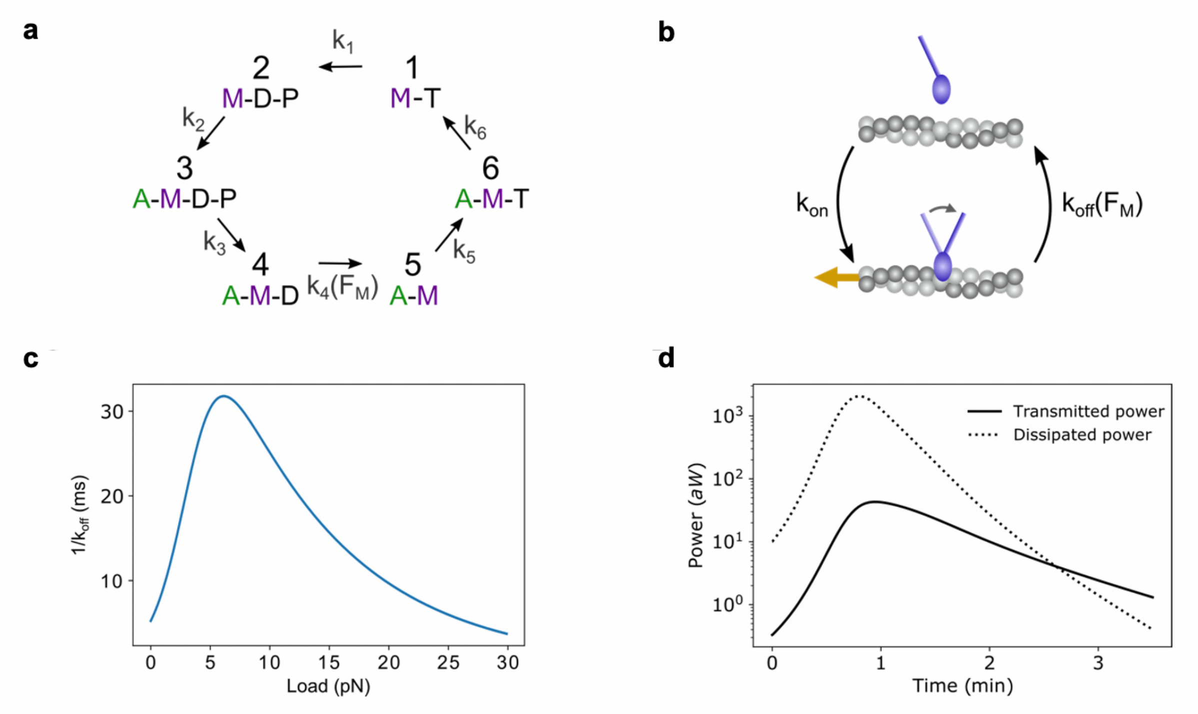

Supplementary Figure 7. Coupling the myosin (un)binding dynamics to the contractility model explains the observed contraction dynamics. a.) The six-step myosin cycle. A = actin, M = myosin, D = ADP, T = ATP and P = phosphate. b.) Simplified cycle with only two states. The load dependent unbinding rate characterizes the catch-slip behavior of the myosin bond. c.) As a consequence, the lifetime of myosin II bonds to F-actin depends on the load on the myosin bond. d.) Estimate of the transmitted and dissipated power of the contraction in Fig. 2i ($k_{\mathrm{p}} = 35\ \mathrm{pN}/\mu\mathrm{m}$) based on our theoretical model.

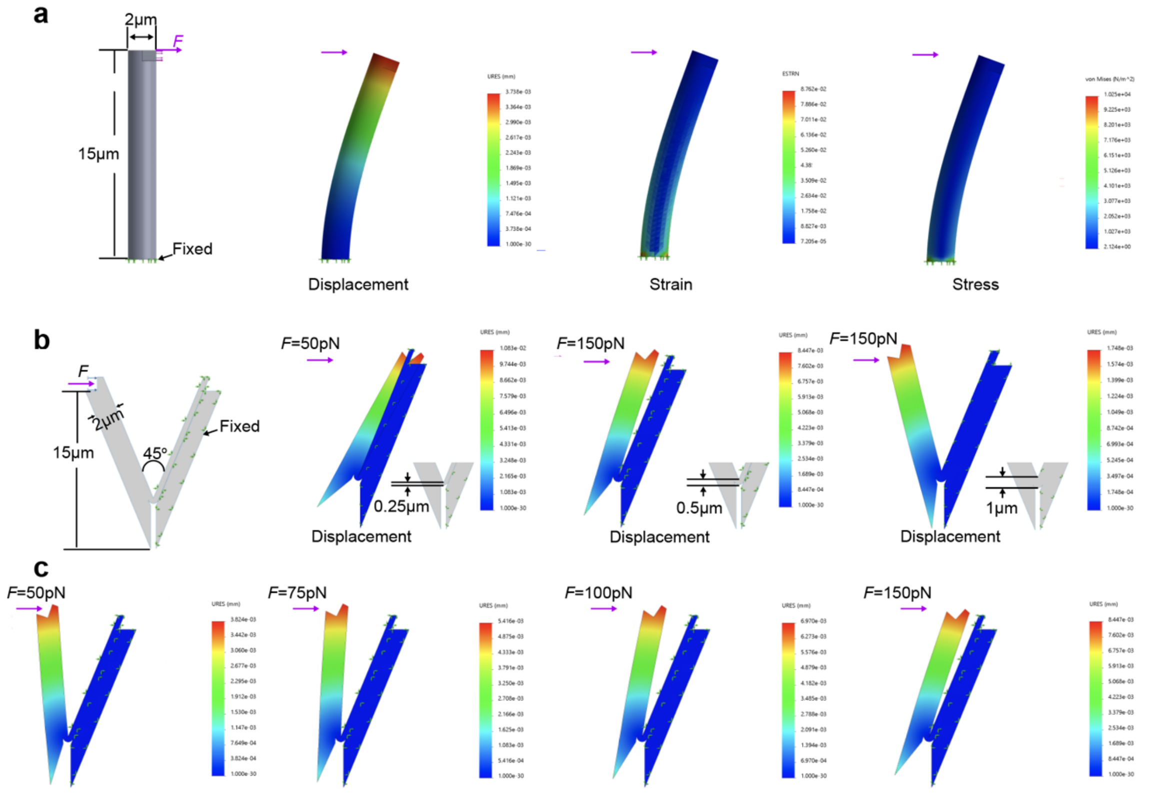

Supplementary Figure 8 Deformations of microstructures under forces simulated by the finite-element model of Solidworks. a.) Schematic illustration and simulation results (displacement, strain and stress) of single pillar that is fixed on the bottom. The applied force on the tip of pillar is 150pN. The displacement of pillar simulated with Solidworks is comparable with the experimental and theoretical results. b.) Design optimization of V-hinges with different thickness of joints. The right arm of hinge is fixed and the force is applied on the tip of another arm. c.) Deformations of V-hinge when applying different forces. URES: resultant displacement; ESTRN: equivalent strain; von Mises: stress. The structural deformations in the presence of external force were simulated using a non-linear model in the commercial finite-element analysis software Solidworks Simulation Premium. For all simulations, the following input parameters were used: the young's modulus 57 kPa and the applied force 150 pN per item. The software setup: geometry nonlinearity options (use large displacement formulation and large strain option); solver (automatic solver selection); incompatible bonding options (more accurate).

Supplementary Tables

Supplementary Table 1. Model parameters

Parameter	Value
x_{catch}	2.5 nm [19]
x_{slip}	0.4 nm [19]
$k_{\text{off}}^{\text{catch}}$	176 s^{-1} [19]
$k_{\text{off}}^{\text{slip}}$	15 s^{-1} [19]
$k_{\text{on}}^{\text{head}}$	10 s^{-1} [22]
k_{on}	0.5 s^{-1}(fitted, Fig. 2i)
a	0.4 (fitted, Fig. 2i)
N_h	28 (fitted, Fig. 2i)
N_{max}	200 (fitted, Fig. 2i)
$h\eta_{\text{am}}^{\text{eff}}$	$6 \cdot 10^3$ µmPas (fitted, Fig. 2i)
$\chi\rho_a(t=0)^2$	0.6 pN/s (fitted, Fig. 2i)
$\rho_a^{\text{max}}/\rho_a(t=0)$	21.7 (fitted, Fig. 2i)

Supplementary Table 2. Contraction energetic for Fig. 2i

Calculated quantity	Value
Dissipated work W_{dis}	$8.4 \cdot 10^{-2}$ pJ
Transmitted work W_{trans}	1.7 pJ

Supplementary Table 3. Comparison of soft actuation methods

Actuation method	Components	Actuation mechanism	Scale	Strain (%)	Strain rate/Speed /response time	Max Force (N)	Control mode/energy source	Sensing	Advantages	Limitations	Ref.
Thermo actuator	SMA	Expansion/ Shrinking	µm-cm	10-50	140mm/s	10^{-1}	External power 30V heating; 2A current	Temperature/light	Easy accessibility; remote control	Uncontrollable on/off; high power consumption; Slow response; less efficiency; limited scalability	23-26
	SMP		µm-cm	50-800	>10s	10^{-1}-10					
	Liquid metal		mm-cm	--	1.2mm/s	--	10-40°C				
	Hydrogel			--	5min-24h	--	>25 °C				
Magnetic actuator	Gel and magnetic particles	Magnetic force, bending	µm-mm	--	<10ms; 2,2mm/s	10^{2}–10^{3}	External power: Magnetic field	Magnetic signals	Controllable on/off; quick response; remote control; high force	Degradation issue; limited scalability; complex manufacturing; auxiliary equipment	23-25
Pneumatic actuator	Fluid based	Contraction, bending, expansion	µm-cm	10-40	10-70%/s	10	Pneumatic pump, valves/air pressure	Pressure	High force densities; Inexpensive	Pressure source required/ large scale	23-25
Photo-actuator		Thermal expansion/contraction	µm-mm	--	0.008-90s	--	Light source > 20mW/cm	Light	Spatiotemporal control; remote control	Low compatibility to bio-tissue; photo-damage; high intensities of light; additional hardware	24,25,27
		Photochemical	µm-mm	--	2.4-60s	10^{-2}-10^{-3}					
Electrical	Ionic Polymer Metal Composites	Bending	µm-cm	0.5-10	1-3%/s	10^{-1}	External power supply(1-5V)	Electrical signals	Controllable on/off	Requirement for wired and bulky; high costs; limited scalability; heat produce	23-25
	Dielectric Elastomer	Contraction, expansion	µm-cm	1-100	10^{2}-10^{5}%/s; 10ms->1s	1	External power: Dielectric field (1-10kV)	Dielectric signals			
Chemicals/nanostructures	Aerogel/	Expansion/shrinking/bending	nm-cm	--	--	10^{-3}	Chemicals supply	Chemicals	Easy accessibility; fast response possible; complex shapes possible	Uncontrollable on/off; temperature dependent; sensitive to environment; slow response	23-25
	pH/chemicals hydrogels			--	1.5-300s	--					
	DNA hydrogel			--	24 hours	--					
Bio-hybrid actuator	Cells/tissue &PDMS	Contraction	µm-mm (100um-8mm)	10-25	38-500µm/s; 10-100%/s	10^{-6}-10^{-3}	Medium	Light Electrical signal	High scalability	Strict maintenance conditions (sterility, oxygen, nutrients, metabolic waste); vascularization required; limited 3D design; high biological complexity; hydration condition	25,28-31
	Engineered motor protein &PDMS	Contraction	mm	--	40s-90s	10^{-6}	Light source: 365nmUV, 0.8W/cm^{-2}, 1-4s.	Light	Minimal bio-design; spatiotemporal control; high scalability	High intensities of light; One shot application; hydration/buffer condition; physically incorporated on soft interface; less robust than cells.	32
**Motor protein**	**Proteins**	**Contraction**	**nm-µm[a]**	**~30**	**0.01-0.1µm/s; 0.2-0.7%/s; 40s-180s**	**10^{-10}-10^{-9}**	**ATP(Catalyzation)**	**Chemicals, light[b]**	**Minimal bio-design; direct energy consumption; untethered; spatiotemporal control; high scalability**	**[a] Limited speed, force and reversibility; energy supply; hydration/buffer condition; less robust than cells**	**This research**

[a] The performances, such as size, force, speed and reversibility can be potentially improved, when the network alignment problem is solved and the automatic microfluidics techniques are introduced.
[b] The sensing signals depend on the implanted bio-sensors.

Supplementary Movies

Supplementary Video 1.

Active contractile dynamics of pillar ring array in response to the motor addition.

Supplementary Video 2.

Active force driving freestanding V-unit folding.

Supplementary Video 3.

Energy sensitive reversible V-unit contraction

Supplementary Video 4.

Unit coordination promoted hydrogel “Zigzag” coiling up.

Supplementary Video 5.

Active force actuating self-folding of hydrogel cubes

Supplementary Video 6.

Bio-actuated 3D protein hydrogel to mimic the grasping microhands

Supplementary Video 7.

Dynamic actions of the robotic arm

Supplementary Video 8.

Sequential and spatiotemporal photo-activation of the artificial arm

References

1 Landau, L. D. & Lifshitz, E. M. Course of Theoretical Physics Vol 7: Theory and Elasticity. in 75–97 (Pergamon press, 1959).

2 Carlsson, A. E. Contractile stress generation by actomyosin gels. *Phys. Rev. E* **74**, 051912 (2006).

3 Ronceray, P. & Lenz, M. Connecting local active forces to macroscopic stress in elastic media. *Soft Matter* **11**, 1597-1605 (2015).

4 Vogel, S. K., Petrasek, Z., Heinemann, F. & Schwille, P. Myosin motors fragment and compact membrane-bound actin filaments. *Elife* **2**, e00116 (2013).

5 Kalganov, A. *et al.* Forces measured with micro-fabricated cantilevers during actomyosin interactions produced by filaments containing different myosin isoforms and loop 1 structures. *Biochim. Biophys. Acta* **1830**, 2710-2719 (2013).

6 Cheng, Y.-S., de Souza Leite, F. & Rassier, D. E. The load dependence and the force-velocity relation in intact myosin filaments from skeletal and smooth muscles. *Am. J. Physiol. Cell Physiol.* **318**, C103-C110 (2020).

7 Kruse, K., Joanny, J.-F., Jülicher, F., Prost, J. & Sekimoto, K. Generic theory of active polar gels: a paradigm for cytoskeletal dynamics. *Eur. Phys. J. E* **16**, 5-16 (2005).

8 Prost, J., Jülicher, F. & Joanny, J.-F. Active gel physics. *Nat. Phys.* **11**, 111-117 (2015).

9 Zemel, A., Rehfeldt, F., Brown, A., Discher, D. & Safran, S. Optimal matrix rigidity for stress-fibre polarization in stem cells. *Nat. Phys.* **6**, 468-473 (2010).

10 Marcq, P., Yoshinaga, N. & Prost, J. Rigidity sensing explained by active matter theory. *Biophys. J.***101**, L33-L35 (2011).

11 Trichet, L. *et al.* Evidence of a large-scale mechanosensing mechanism for cellular adaptation to substrate stiffness. *Proc. Natl. Acad. Sci. U. S. A.* **109**, 6933-6938 (2012).

12 Mizuno, D., Tardin, C., Schmidt, C. F. & MacKintosh, F. C. Nonequilibrium mechanics of active cytoskeletal networks. *Science* **315**, 370-373 (2007).

13 Landau, L. & Lifshitz, E. Course of Theoretical Physics Vol 6: Fluid mechanics. in 44–49 (Pergamon Press Oxford, 1987).

14 Bendix, P. M. *et al.* A quantitative analysis of contractility in active cytoskeletal protein networks. *Biophys. J.* **94**, 3126-3136 (2008).

15 Joanny, J.-F., Kruse, K., Prost, J. & Ramaswamy, S. The actin cortex as an active wetting layer. *Eur. Phys. J. E* **36**, 1-6 (2013).

16 Foster, P. J., Fürthauer, S., Shelley, M. J. & Needleman, D. J. Active contraction of microtubule networks. *Elife* **4**, e10837 (2015).

17 Chugh, P. *et al.* Actin cortex architecture regulates cell surface tension. *Nat. Cell Biol.* **19**, 689-697 (2017).

18 Koenderink, G. H. *et al.* An active biopolymer network controlled by molecular motors. *Proceedings of the National Academy of Sciences* **106**, 15192-15197 (2009).

19 Guo, B. & Guilford, W. H. Mechanics of actomyosin bonds in different nucleotide states are tuned to muscle contraction. *Proc. Natl. Acad. Sci. U. S. A.* **103**, 9844-9849 (2006).

20 Erdmann, T., Albert, P. J. & Schwarz, U. S. Stochastic dynamics of small ensembles of non-processive molecular motors: The parallel cluster model. *J. Chem. Phys.* **139**, 11B604_601 (2013).

21 Van Kampen, N. G. Stochastic processes in physics and chemistry. in vol. 1 298–301 (Elsevier, 1992).

22 White, H. D., Belknap, B. & Webb, M. R. Kinetics of nucleoside triphosphate cleavage and phosphate release steps by associated rabbit skeletal actomyosin, measured using a novel fluorescent probe for phosphate. *Biochemistry* **36**, 11828-11836 (1997).
23 Sinibaldi, E., Argiolas, A., Puleo, G. L. & Mazzolai, B. Another lesson from plants: the forward osmosis-based actuator. *PloS one* 9, e102461 (2014).
24 Hines, L., Petersen, K., Lum, G. Z. & Sitti, M. Soft actuators for small-scale robotics. *Adv. Mater.* 29, 1603483 (2017).
25 Rich, S. I., Wood, R. J. & Majidi, C. Untethered soft robotics. *Nat. Electron.* 1, 102-112 (2018).
26 El-Atab, N. et al. Soft Actuators for Soft Robotic Applications: A Review. *Adv. Intell. Syst.* 2, 2000128 (2020).
27 Chen, Y. et al. Light-Driven Bimorph Soft Actuators: Design, Fabrication and Properties. *Mater. Horiz*. (2020). doi.org/10.1039/D0MH01406K
28 Feinberg, A. W. Biological soft robotics. *Annu. Rev. Biomed. Eng.* 17, 243-265 (2015).
29 Chan, V., Asada, H. H. & Bashir, R. Utilization and control of bioactuators across multiple length scales. *Lab Chip* 14, 653-670 (2014).
30 Raman, R., Cvetkovic, C. & Bashir, R. A modular approach to the design, fabrication, and characterization of muscle-powered biological machines. *Nat. Protoc.* 12, 519-533 (2017).
31 Guix, M. et al. Biohybrid soft robots with self-stimulating skeletons. *Sci. Robot* 6, eabe7577, doi:10.1126/scirobotics.abe7577 (2021).
32 Nitta, T., Wang, Y., Du, Z., Morishima, K. & Hiratsuka, Y. A printable active network actuator built from an engineered biomolecular motor. *Nat. Mater.*, 1-7 (2021).
