# Supplementary material for: 3D printed protein-based robotic structures actuated by molecular motor assemblies
Source: Nat Mater. 2022 May 26;21(6):703–9. doi: 10.1038/s41563-022-01258-6 (PMC9156402; doi:10.1038/s41563-022-01258-6)
Supplement: Supplementary file 2 — Reporting Summary [file 41563_2022_1258_MOESM2_ESM.pdf]

nature portfolio

Corresponding author(s): Petra Schwille and Chase Broedersz

Last updated by author(s): Mar 14, 2022

Reporting Summary

Nature Portfolio wishes to improve the reproducibility of the work that we publish. This form provides structure for consistency and transparency in reporting. For further information on Nature Portfolio policies, see our Editorial Policies and the Editorial Policy Checklist.

Statistics

For all statistical analyses, confirm that the following items are present in the figure legend, table legend, main text, or Methods section.

n/a	Confirmed	
☐	☒	The exact sample size (*n*) for each experimental group/condition, given as a discrete number and unit of measurement
☐	☒	A statement on whether measurements were taken from distinct samples or whether the same sample was measured repeatedly
☐	☒	The statistical test(s) used AND whether they are one- or two-sided<br>*Only common tests should be described solely by name; describe more complex techniques in the Methods section.*
☒	☐	A description of all covariates tested
☒	☐	A description of any assumptions or corrections, such as tests of normality and adjustment for multiple comparisons
☐	☒	A full description of the statistical parameters including central tendency (e.g. means) or other basic estimates (e.g. regression coefficient) AND variation (e.g. standard deviation) or associated estimates of uncertainty (e.g. confidence intervals)
☐	☒	For null hypothesis testing, the test statistic (e.g. *F*, *t*, *r*) with confidence intervals, effect sizes, degrees of freedom and *P* value noted<br>*Give P values as exact values whenever suitable.*
☒	☐	For Bayesian analysis, information on the choice of priors and Markov chain Monte Carlo settings
☒	☐	For hierarchical and complex designs, identification of the appropriate level for tests and full reporting of outcomes
☒	☐	Estimates of effect sizes (e.g. Cohen's *d*, Pearson's *r*), indicating how they were calculated

Our web collection on statistics for biologists contains articles on many of the points above.

Software and code

Policy information about availability of computer code

Data collection	Zeiss Zen software (Zen 2.1 black), ZeissZen 2.6 (Blue edition) , SPM(JPK NanoWizard software) and TESCAN MIRA3 control software(4.2.32.0 build 1556) were used to acquire images.<br>Zeiss Zen software (Zen 2.1 black): Fig. 2(d, e, h), Fig. 3(b-c), Fig. 4(b, d, f, g, i), E.D. Fig. 2 f, E.D. Fig. 3 (a, c, e-h), E.D. Fig. 4 (b-d), E.D. Fig. 5 (e-f), E.D. Fig. b (a, b, d). E.D. Fig. 9 (a, e-f) and E.D. Fig. 10 (a-c). ZeissZen 2.6 (Blue edition): Fig. 2b and E.D. Fig. 5c and Supplement Fig. 3.<br>TESCAN MIRA3 control software(4.2.32.0 build 1556): E.D. Fig. 1 (a-d). SPM(JPK NanoWizard software) : E.D. Fig. 2 (a, b, d)
Data analysis	1. Zeiss Zen software (Zen 2.1 black): Fig. 4(d, f, g, i), E.D. Fig. 5e-f.<br>2. ImageJ 1.53f51(Fiji): Fig. 2(d, e, h), Fig. 3(b, c), Fig. 4(b, d, f, g, i), E.D. Fig. 2(a, f), E.D. Fig. 3(a, c-h), E.D. Fig. 4(b-d), E.D. Fig. 5c, E.D. Fig. 8(a-b, d), E.D. Fig. 9, E.D. Fig. 10 a-c, Supplement Fig. 3.<br>3. Origin2019b: Fig. 2(c, i, j), Fig. 3(c_curve, d), E.D. Fig. 2(c, e), E.D. Fig. 3(b, d), E.D. Fig. 4(f-g), E.D. Fig. 5(b, d), E.D. Fig. 6, E.D. Fig. 7, E.D. Fig. 8e, E.D. Fig. 10 (d-e), and Supplement Fig. 1,<br>4. JPK data processing software Version5.1.4:E.D. Fig. 2(b-e).<br>5. Solidworks2019: Supplement Fig. 8).<br>6. Matlab R2016a and Python 3.7: Fig. 2(f, g), Supplementary Fig.5, Supplementary Figure. 6 and Supplement Fig. 7. Our custom code can be found at https://github.com/JFlommersfeld/Actomyosin-contractions-in-soft-pillar-rings.

For manuscripts utilizing custom algorithms or software that are central to the research but not yet described in published literature, software must be made available to editors and reviewers. We strongly encourage code deposition in a community repository (e.g. GitHub). See the Nature Portfolio guidelines for submitting code & software for further information.

Data

Policy information about availability of data

All manuscripts must include a data availability statement. This statement should provide the following information, where applicable:

- Accession codes, unique identifiers, or web links for publicly available datasets
- A description of any restrictions on data availability
- For clinical datasets or third party data, please ensure that the statement adheres to our policy

All data used in this paper are available at Figshare with the identifier https://doi.org/10.6084/m9.figshare.19345874.v1 or from the corresponding authors upon request. Source data are provided with this paper.

Field-specific reporting

Please select the one below that is the best fit for your research. If you are not sure, read the appropriate sections before making your selection.

☒ Life sciences ☐ Behavioural & social sciences ☐ Ecological, evolutionary & environmental sciences

For a reference copy of the document with all sections, see nature.com/documents/nr-reporting-summary-flat.pdf

Life sciences study design

All studies must disclose on these points even when the disclosure is negative.

Sample size	Samples sizes were limited by practicality and throughput, with a minimum requirement of at least three independent experiments.
Data exclusions	Images were only excluded from analysis when the printed micro-structures were defective after developing.
Replication	The numbers of experimental repeats are indicated in the respective figure legends or in the method section of Statistics and Reproducibility. All observations were replicated successfully.
Randomization	Sample were mixed from scratch. No allocation is done and randomization is not applicable, due to the type of experiments performed and the nature of the study.
Blinding	Blinding was not required, since no data are excluded, except for the defective 3D printed structures. And all analysis was intrinsically without user-bias.

Reporting for specific materials, systems and methods

We require information from authors about some types of materials, experimental systems and methods used in many studies. Here, indicate whether each material, system or method listed is relevant to your study. If you are not sure if a list item applies to your research, read the appropriate section before selecting a response.

Materials & experimental systems

n/a	Involved in the study	
☒	☐	Antibodies
☒	☐	Eukaryotic cell lines
☒	☐	Palaeontology and archaeology
☒	☐	Animals and other organisms
☒	☐	Human research participants
☒	☐	Clinical data
☒	☐	Dual use research of concern

Methods

n/a	Involved in the study	
☒	☐	ChIP-seq
☒	☐	Flow cytometry
☒	☐	MRI-based neuroimaging
